# Considerations and Implications in the Purification of Extracellular Vesicles – A Cautionary Tale

**DOI:** 10.3389/fnins.2019.01067

**Published:** 2019-10-18

**Authors:** Yi Xin Fiona Lee, Henrik Johansson, Matthew J. A. Wood, Samir El Andaloussi

**Affiliations:** ^1^Department of Physiology, Anatomy and Genetics, University of Oxford, Oxford, United Kingdom; ^2^Genome Institute of Singapore, Agency for Science, Technology and Research, Singapore, Singapore; ^3^Cancer Proteomics Mass Spectrometry, Science for Life Laboratory, Department of Oncology-Pathology, Karolinska Institutet, Stockholm, Sweden; ^4^Department of Laboratory Medicine, Karolinska Institutet, Stockholm, Sweden

**Keywords:** extracellular vesicles, stem cells, proteomics, exosomes, isolation, purification

## Abstract

Extracellular vesicles (EVs) are nano-sized particles constitutively released from cells into all biological fluids. Interestingly, these vesicles contain genetic cargoes including proteins, RNA and bioactive lipids that can be functionally delivered and affect recipient cells. As a result, there is growing interest in studying EVs in pathological conditions, including central nervous system (CNS)-related diseases, as EVs may be used for diagnostic purposes or as therapeutic agents. However, one major bottleneck is the need for better EV purification strategies when considering complex biological sources such as serum/protein-rich media or plasma. In this study, we have performed a systematic comparison study between the current gold-standard method: ultracentrifugation, to an alternative: size-exclusion chromatography (LC), using induced pluripotent stem cell (iPSC) derived complex media as a model system. We demonstrate that LC allows for derivation of purer EVs from iPSCs, which was previously impossible with the original UC method. Importantly, our study further highlights the various drawbacks when using the conventional UC approach that lead to misinterpretation of EV data. Lastly, we describe novel data on our iPSC-EVs; how they could relate to stem cell biology and discuss their potential use as EV therapeutics for CNS diseases.

## Introduction

The very first report on secretory material from cells was by Wolf in 1967; here he observed the presence of minute phospholipid-rich, pro-coagulant substance from fresh platelet-free plasma which he then termed as “platelet-dusts” (Wolf, 1967). Subsequently, more studies revealed that these “dusts” were actually membranous nanosized particles that contained functional molecules, had downstream effects on recipient cells and could be collected from various bodily fluids (Raz et al., 1978; Trams et al., 1981). Based on the state of cells and mechanisms of release, these particles can be broadly classified into three main populations: “exosomes”- a pool of vesicles originating from the multivesicular endosome; “microvesicles”- a heterogeneous set of particles which directly shed off the plasma membrane, and “apoptotic bodies”- a much larger group of micelles which bleb off dying cells undergoing apoptosis (Harding et al., 1984; Pan et al., 1985; Raposo et al., 1996). Despite such known differences, there still appears to be significant overlaps in both molecular composition and size across all three populations. As it is challenging to purify each individual group, there has been an active shift to use “extracellular vesicles (EVs)” as a collective term to instead describe these secretory vesicles (Bobrie et al., 2012; Gould and Raposo, 2013).

### EVs as Communicators of Physiology and Disease

Historically, EVs were postulated to be the “garbage trucks” of cells; helping to remove unwanted molecular material out of cells. However, there are an increasing number of studies describing dedicated protein and RNA machineries that regulate the sorting of specific molecular contents (proteins and RNA) into and onto these vesicles (Szostak et al., 2014; Villarroya-Beltri et al., 2014; Janas et al., 2015; Hung and Leonard, 2016; Ragusa et al., 2017; Moreno-Gonzalo et al., 2018; Sork et al., 2018). Subsequently, these EVs serve as delivery men by following the unique “addresses” on their surface. Thus, EVs ferry their well-protected molecular cargoes within the bi-lipid boundaries and elicit phenotypic effects on recipient targets, some of which may locate at distal sites from their origin.

The presence and involvement of EVs in biology is best understood in the areas of immunology and cancer. Notably, EVs from dendritic cells can mimic their parental source to aid in antigen presentation and elicit a T cell response in normal physiological processes (Raposo et al., 1996; Zitvogel et al., 1998; Théry et al., 2001; Utsugi-Kobukai et al., 2003; Segura et al., 2005a, b). However, not all EV-mediated communications are beneficial, as in disease settings EVs can aid in the spread of pathogenesis. For example, EVs from primary cancer cells assist in the preparation of the pre-metastasis niche for colonization of migratory cancer cells (Janowska-Wieczorek et al., 2005; Peinado et al., 2012). Interestingly, one study found that subpopulations of EVs are secreted by a single pool of breast cancer cells and that each EV subtype can influence the formation of micro-metastases in the different organs, depending on the types of surface integrins (Hoshino et al., 2015).

### EVs Contribute to Intercellular Brain Communication

The brain is a complex organ consisting of myriad neural networks between the multitudes of cell types. Hence, it is no surprise that there has been a steadily increasing body of evidence showing how EVs contribute to normal brain function and in central nervous system (CNS)-related diseases (Thompson et al., 2016). As in other body systems, all cell types of the brain, including neurons, oligodendrocytes, astrocytes and microglial cells, release EVs (Fauré et al., 2006; Krämer-Albers et al., 2007; Antonucci et al., 2012; Wang et al., 2012). Depending on the cell source and the physiological state, the characteristics of secreted EVs can vary accordingly and induce different functions in the brain. For example, EVs from neurons present the AMPA receptor subunit GluR2 and this contributes to synapse maturation and plasticity (Lachenal et al., 2011). Alternatively, EVs from neurons are enriched in syndecans and tetraspanins (e.g., CD81), which are important signaling cues (Bahi and Dreyer, 2005; Hienola et al., 2006). Hence, EVs can assist in neurite outgrowth and axon path finding. In other instances, EVs can act as important tools for the cross talk between different cell types in the brain, such as when oligodendrocytes release EVs in response to neuronal activity. These EVs carry metabolites such as catalase, which can be delivered back to the neurons for trophic support (Frühbeis et al., 2013). Similarly, Schwann cells have been shown to release EVs upon axonal damage (Krämer-Albers et al., 2007), where they could be active at the injury site and aid in axonal regeneration.

### Technical Issues in EV Research-Purification Strategies

Despite growing knowledge of the importance of EVs to multicellular functions, there is still a lack of consensus on the methodologies for purifying EVs from cells. To date, the most common strategy remains differential ultracentrifugation (UC) due to its accessibility in most laboratories (Théry et al., 2006). However, with improved technologies for EV phenotyping, it is becoming increasingly evident that this “gold-standard” UC protocol suffers from several drawbacks; most of which are related to the yield, purity and physical integrity of EVs (Cvjetkovic et al., 2014; Nordin et al., 2015).

To address this issue, alternative methods for EV purification have been exploited. These include precipitation-based strategies; some of which can be coupled with antibody-capture methods on microfluidic devices or the use of magnetic nanowires (Ghosh et al., 2014; Kanwar et al., 2014; Lim et al., 2019; Sunkara et al., 2019). However, these methods may not be scalable, often compromise on EV purity (Yamada et al., 2012) and require more studies on if the purified EVs can be efficiently eluted and remain complementary to use for any downstream functional characterization. One other method which has gained popularity in recent years is size-exclusion liquid chromatography (LC). Unlike UC, the LC process does not require high speed spins and is scalable for large volume samples when combined with a pre-concentration step. All in all, many supporting reports have verified the feasibility of using LC for EV purification in several serum-free cell culture-based systems and in biological fluids (Chen et al., 2011; Arslan et al., 2013; Böing et al., 2014; Nordin et al., 2015; Welton et al., 2015; Mol et al., 2017; Oeyen et al., 2018; Lane et al., 2019; Takov et al., 2019).

Generally in EV research, it is required to use serum-free or exosome-cleared FBS for collection of conditioned media, as regular serum contains numerous microvesicles that can contaminate and confound the downstream analysis of the intended cellular EVs (Noerholm et al., 2012; Shelke et al., 2014; Wei et al., 2016; Kornilov et al., 2018; Lehrich et al., 2018). However, this becomes an issue when considering cell types which will change in serum-depleted media, such as stem cells. In all the previously studies on protocol comparison (Rood et al., 2010; Alvarez et al., 2012; Tauro et al., 2012; Rekker et al., 2014; Zhang et al., 2014; Livshits et al., 2015; Stranska et al., 2018; Takov et al., 2019), none have compared purification of EVs from protein-rich, complex media sources such as stem cell media. Here, we describe a systematic comparison study between UC and LC purified EVs from iPSCs. Interestingly, we find that their molecular contents vary based on protocol, which will have implications on the interpretation of EV content and functionality. Lastly, we cross compared EVs to their parental sources and discuss the feasibility of using these iPSCs for CNS related research.

## Materials and Methods

### Cell Culture of Pluripotent Stem Cell Lines and Embryonic Fibroblasts

Mouse embryonic fibroblasts (MEFs, derived from Dr. Paul Fairchild’s lab) were grown in complete MEF growth media comprising of DMEM (Life Technologies) supplemented with 15% FBS and 50 μg/ml Penicillin/Streptomycin (P/S). To prepare feeders for stem cell culture, MEFs were treated with mitomycin-C (Sigma, United Kingdom) at 1 mg/ml of MEF media for 2 h. The treated MEFs were then washed three times with PBS and re-plated on fresh culture flasks. Mouse stem cell lines [embryonic stem cells (ESCs): ESF121, ESF116 and induced pluripotent stem cells (iPSCs): iMEF14, iMEF19]; all derived from Dr. Paul Fairchild’s lab (Fairchild et al., 2000) were first cultured on mitomycin-C treated MEFs feeders. When the cells reached 80% confluence, one-tenth of the cells were plated on fresh feeders while the remainder were plated on 0.1% gelatin-coated plates. For both feeder and feeder-free conditions, cells were cultured in complete mouse stem cell media comprised of DMEM (Lonza, United Kingdom) supplemented with 15% knockout serum-replacement supplement (KOSR) (Life Technologies, United Kingdom), 2 mM L-glutamine (Life Technologies), 1 mM sodium pyruvate (Life Technologies), 0.1 mM non-essential amino acids (Life Technologies), 50 (μg/ml of P/S, 0.2 mM 2-mercaptoethanol (Sigma) and 106 units of mouse leukaemia inhibitory factor (mLIF, Miltenyi Biotec, United Kingdom). All cells were cultured at 37°C with 5% CO_2_.

### Collection of Conditioned Media (CM) for EV Purification

For the initial data shown in Supplementary Figure S1, pre-spun stem cell (PS) media described here was complete stem cell media that had been pre-spun at 120,000 *g* for 16 h prior to use. The OptiMEM (OM) media was OptiMEM (Life Technologies) supplemented with 50 μg/ml of P/S. For both ESC and iPSC cell lines, the stem cells were cultured twice on 0.1% gelatin-coated plates to get rid of any contaminating feeder cells prior to use for EV collection. For each cell line, 1M cells were initially seeded on a single 10 cm plate. When cells reached 70% confluence (48 or 72 h after plating), the growth media was removed; cells were washed with PBS and replaced with fresh stem cell media, PS or OM depending on the experimental set-up. Conditioned media (CM), ranging from 150–200 ml, was then collected 48 h after media change; cells were harvested by trypsin and counted with a hemocytometer. For each collection, the total CM was first centrifuged at 300 *g*, 5 min to get rid of cellular debris. The supernatant was decanted and further centrifuged at 2,000 *g*, 10 min to get rid of larger particles before subjecting to filtration through a 0.22 μm syringe filter. Subsequently, the CM was split equally into two volumes for UC and LC purification concurrently.

### Purification of EVs

#### Purification of EVs by Differential Centrifugation Protocol (UC)

The UC protocol used for purification of EVs is based on an established protocol described by Théry et al. (2006). Briefly, the filtered CM was spun at 120,000 *g*, 70 min to pellet EVs. To eliminate any protein contaminations, the pellet was re-suspended in 25 ml of PBS and spun again at 120,000 *g* for 70 min. All centrifugation steps were performed at 4°C. The resultant pellet was then re-suspended in 100 μl of PBS and used fresh for analysis or kept at −80°C for further proteomics analysis.

#### Purification of EVs by Size-Exclusion Liquid Chromatography Protocol (LC)

The LC protocol used for purification of EVs was based on the method described by Taylor et al. (2011), with some slight modifications. Briefly, the filtered CM was concentrated using the Amicon 100k-Da molecular weight cut-off (MWCO) filters (Millipore, United Kingdom) at 3,500 *g* for 15 min. The concentrate retentate was then loaded onto a Sephacryl S-400 16/60 LC column (GE Healthcare, Sweden) and run with PBS at 0.5 ml/min. Fixed-volume 2 ml fractions of the eluted solutions were then collected with a fraction collector. Based on the 280 nm LC chromatograph, fractions covering the first 280 nm LC peak, designated as “F1,” were pooled and concentrated with Amicon 10-kDa MWCO filters (Millipore) at 3,500 *g* for 15 min down to 100 μl of PBS and used fresh for analysis or kept at −80°C for further proteomics analysis. All centrifugation and LC processes were done at 4°C.

### Sucrose Gradient Density Centrifugation of EVs

The sucrose gradient density centrifugation protocol used here was based from the protocol described by Théry et al. (2006). Briefly, hydroxyethylpiperazine-*N*′-2-ethanesulfonic acid (HEPES)/sucrose stock solution was prepared by mixing 428 g of protease-free sucrose (Sigma) in 500 ml of 20 mM of HEPES buffer. The pH was adjusted to 7.4 with 1M sodium hydroxide (NaOH) and stored at 4°C. Prior to construction of the sucrose gradient, the HEPES/sucrose stock solution was diluted with 20 mM HEPES buffer to generate 10 concentrations of sucrose solutions (0.25–2.5M with 0.25 increments). The EV sample collected after UC and LC was pre-mixed with either 0.25 or 2.5 M of HEPES/sucrose stock to 1 ml total volume and loaded at either at the top or bottom of the linear sucrose gradient respectively. The sucrose gradient was then centrifuged at 200,000 *g* for 16 h or 72 h at 4°C in a SW 40 swing rotor (Beckman Coulter). One ml fractions were carefully collected from the top and each fraction was weighed to obtain an estimated density of each fraction. Each 1ml sucrose fraction was subsequently diluted in 25 ml of PBS and centrifuged at 120,000 *g* for 70 min at 4°C to wash and pellet the particles. The resultant UC pellet of each fraction was re-suspended in 50 μl of PBS and subjected to molecular analysis.

### Quantification and Characterization of EVs

#### Nanoparticle Tracking Analysis (NTA)

NTA allows for the quantification of total particle amounts and size distribution of particles based on Brownian motion of particles. All NTA was done with the NTA2.3 software on the NS500 Nanosight machine (Nanosight, Malvern, United Kingdom). Before each run, the NS500 measurements were calibrated with known concentrations of 100 nm silica microspheres to obtain optimum acquisition detector settings and post-acquisition settings. For all our recordings, we used a camera level of 14 (shutter speed 600, camera gain 250) and automatic function for all post-acquisition settings: detection threshold level 5, blur and minimum expected particle size. EV samples were diluted in PBS prior measurement, starting at an initial dilution of 1:100, and then further adjusted for each sample individually to achieve a particle count of between 2 × 10^8^ per ml to 1 × 10^9^ per ml. Once the dilution of the sample was determined, the sample was loaded in the sample chamber and the camera focus was adjusted to make the particles appear as sharp dots of light. Using the *script control* facility on the NTA2.3 software, we recorded five 30 s videos for each sample; incorporating a sample advance and 5 s delay between each recording. The measurements were then analyzed using the *batch process* facility and results were exported as Microsoft Excel spreadsheets for further analysis. If the profiles were not in agreement, measurements were then repeated.

### Protein Quantification of EVs and Cell Lysates

Extracellular vesicles and cell lysates were quantified using the microBCA or the BCA assay kit (Thermo Scientific Fisher) respectively as indicated by the manufacturer’s instructions.

### Western Blotting (WB)

Depending on the experimental set-up, either a fixed volume or a set number of particles (as calculated by NTA) from the re-suspension of EV pellet was used. For cells, after trypsin treatment, cells were collected with PBS and spun at 1,500 *g*, 5 min to pellet cells. Cells were washed in PBS and pellet at 1,500 *g*, 5 min again. The supernatant was decanted and the cell pellet was lysed in radioimmunoprecipitation assay (RIPA) buffer for 1 h at 4°C. The mixture was then spun at 16,000 *g*, 20 min. The supernatants were then measured for protein concentration and fixed proteins amounts were used for WB. The EV/cell sample was mixed with 2x Laemilli sample buffer (Bio-Rad, United Kingdom) containing 5% β-mercaptanol and heated at 100°C for 10 min. Samples were then spun-down briefly before being loaded in 1.5 mm, 12% home-made Tris/Glycine SDS-polyacrylamide gels and ran at 170 V for 70 min in running buffer, until the dye front reaches the bottom of the tank. Proteins on the gel were transferred to a polyvinylidene fluoride (PVDF) membrane (Millipore) at 100 V for 70 min in transfer buffer containing 20% methanol. Membranes were then incubated in blocking buffer [5% fat free milk in Tris Buffer Saline with 0.1% Tween-20 (TBS-T, Sigma)] for 60 min at room temperature (RT) on a rocker with gentle shaking. After blocking, the membrane was incubated with freshly prepared primary antibody solution [from Abcam, United Kingdom: anti-Alix, ab117600; anti-Tsg101, ab30871; anti-CD9, ab92726; anti-Calnexin, ab22595; anti-Oct4, ab19857, anti-β-actin, ab8268, all used at 1:1000; from Santa Cruz: anti-CD81(H-121), sc-9158 used at 1:100] overnight at 4°C or 2 h at RT. Membranes were then washed three times 10 min each using washing buffer (TBS-T) with vigorous shaking before adding the secondary antibody solution [all from LiCOR, United Kingdom: Goat anti-mouse IgG IRDye^®^ 800CW (925-32210) and 680RD (925-68070); Goat anti-rabbit IgG IRDye^®^ 800CW (925-32211) abd 680RD (925-68071), all used at 1:10000] and incubated for 2 h at RT. After secondary incubation, membranes were washed three times 10 min each with TBS-T and visualized by scanning both 700- and 800 nm channels on the LI-COR Odyssey CLx infrared imaging system. For re-probing on the same membrane, the membrane was first washed three times 10 min each before re-incubation with the next primary antibody.

### Transmission Electron Microscopy (TEM)

A 200 mesh nickel carbon/formvar grid (AgarScientific) was placed onto a 10 μl droplet of the EV suspension for 15 min. The grid was then blotted dry with filter paper, immediately transferred to a 15 μl droplet of 2% uranyl acetate for 1 min and protected from light. The grid was again blotted dry with filter paper before being transferred to a 15 μl droplet of filtered distilled and deionized water (ddH_2_0) for 1 min. The grid was then blotted dry and left to air dry on the bench top for 15 min. EVs negatively stained on this grid was then visualized with a JEOL 1010 transmission electron microscope (JEOL, Tokyo, Japan).

### Trizol Extraction of RNA From EVs and Cells

Total RNA from EVs and cells were extracted based on the manufacturer’s protocol. Briefly, 75 μl of the EV suspension or 2 × 10^5^ cells were mixed in Trizol LS (Life Technologies) and incubated for 5 min at RT. Sixty μl of chloroform was then added and tubes were shaken for 15 s. The mixed samples were then incubated for 15 min at RT before being centrifuged at 12,000 *g* for 15 min at 4°C to derive the three distinct phases. The upper colorless phase was transferred to a new tube and 150 μl of isopropanol with 1 μl of glycogen was added. The sample was vortex briefly and incubated at RT for 10 min. The sample was then centrifuged at 12,000 *g* for 10 min at 4°C and the supernatant were discarded. The remaining white RNA pellet was washed with 300 μl of 75% ethanol and then spun down at 7,500 *g* for 5 min at 4°C. The ethanol was then discarded, and the pellet was air-dried for 5–10 min, till it turned transparent, and then re-dissolved in 20 μl of RNase-free water. The mixture was then incubated at 55–60°C for 15 min on a heat block. RNA concentrations of the samples were then measured using the Quant-iT^TM^ RiboGreen^®^ RNA Assay Kit (Life Technologies).

### RNA Profiling With Agilent Bioanalyzer Software

Quality and size of the EV and cellular RNA were detected using capillary electrophoresis with the Agilent RNA 6000 Pico kit and Agilent RNA small RNA kit on an Agilent 2100 Bioanalyzer^®^ (Agilent Technologies, Santa Clara, CA, United States) according to the manufacturer’s protocol.

### LC/MS/MS Proteomic Analysis

As both mouse iPSCs and ESCs were derived off the same strain background (CBA/Ca) and derived using the same methodology, we proceeded with proteomics analysis on one of the miPSC line- iMEF14 and one of the mESC line- ESF121. For each cell and EV sample, three biological replicates were prepared and analyzed (Supplementary Table S1). All cells and EVs were concentrated by speedvac and lysed with 1% SDS, 25 mM HEPES, 1 mM DTT. Lysates were heated to 95°C for 5 min followed by sonication for 1 min and centrifugation at 14,000 *g* for 15 min. The supernatant was mixed with 1 mM DTT, 8 M urea, 25 mM HEPES, pH 7.6 and transferred to a 10-kDa cut-off centrifugation filtering unit (Pall, Nanosep^®^), and centrifuged at 14,000 *g* for 15 min, followed by an addition of the 8 M urea buffer and centrifugation again. Proteins were alkylated by 50 mM iodoacetamide (IAA) in 8 M urea, 25 mM HEPES for 10 min, The proteins were then centrifuged at 14,000 *g* for 15 min followed by 2 more additions and centrifugations with 8 M urea, 25 mM HEPES. Trypsin (Promega) in 250 mM urea, 50 mM HEPES was added to the cell lysate at a ratio of 1:50 trypsin:protein and incubated overnight at 37°C. The filter units were centrifuged at 14,000 *g* for 15 min followed by another centrifugation with milli-Q water (MQ) and the flow-through was collected. Peptides were cleaned by a strata-X-C-cartridge (Phenomenex).

Before analysis on the Q Exactive (Thermo Fischer Scientific, San Jose, CA, United States), peptides were separated using an Agilent 1200 nano-LC system. Samples were trapped on a Zorbax 300SB-C18, and separated on a NTCC-360/100-5-153 (Nikkyo Technos., Ltd.) column using a gradient of A [3% acetonitrile (ACN), 0.1% formic acid (FA)] and B (95% ACN, 0.1% FA), ranging from 7 to 40% B in 240 min with a flow of 0.4 μl/min. The Q Exactive was operated in a data dependent manner, selecting top 5 precursors for fragmentation by HCD. The survey scan was performed at 70,000 resolution from 300–1700 *m/z*, using lock mass at *m/z* 445.120025, with a max injection time of 100 ms and target of 1 × 10^6^ ions. For generation of HCD fragmentation spectra, a max ion injection time of 500 ms and AGC of 1 × 10^5^ were used before fragmentation at 30% normalized collision energy, 17,500 resolution. Precursors were isolated with a width of 2 *m/z* and put on the exclusion list for 70 s. Single and unassigned charge states were rejected from precursor selection.

Proteome discoverer 1.3 with sequest-percolator was used for protein identification. Precursor mass tolerance was set to 10 ppm and for fragments to 0.02 Da. Oxidized methionine and was set as dynamic modification, and carbamidomethylation as static modification. Spectra were matched to a combined *Mus musculus* and *Bos taurus* ensembl 72 database, and results were filtered to 1% FDR. Identifications in *Bos taurus* was considered to originate from FBS and removed. For this set of LC-MS/MS, the reference list used was generated based on all proteins identified within this stem cell set. In this proteomics run, only a short gradient of the cellular and vesicular proteome was subjected to analysis. Hence, the overall proteome of the cell described here may only recapitulate a portion of the total proteome as expected. For classification of commonly identified proteins when comparing two sample types, we calculated the ratio of area of proteins of one sample over the other. Proteins that had a twofold change in ratio were designated to be “up-regulated.” These groups were then analyzed in the online platform: Panther to evaluate enrichment of Gene ontology (GO) (Mi et al., 2013, 2019).

### Statistics

The Student’s *t*-test was used when comparing EVs derived by the UC versus LC purification.

## Results

### Alternative Forms of Serum-Depleted Media Not Feasible for Stem Cells

In our study, mouse induced pluripotent stem cells (iPSCs) were used as a model for sensitive cells that require complex media types for their cultivation. Unlike differentiated cells, serum is crucial for the maintenance of stem cells in their undifferentiated state. However, in EV research, the use of serum is problematic as serum contains microvesicles, which could be co-purified and interfere with the downstream analysis of EVs. Hence, we used knockout serum replacement (KOSR), which is devoid of serum microvesicles. However, KOSR could still contain other undefined components, which could confound the EV purity.

To evaluate the background particulate counts in KOSR-supplemented (SR) media, we applied the UC purification protocol to unconditioned media and analyzed the pellet. In parallel, two other media types commonly used in EV research were compared; pre-spun SR-media (PS) and serum-free OptiMEM (OM). Interestingly, NTA measurements indicated the presence of nanoparticles in all pellets, most significantly in the SR-media pellet (Supplementary Figures S1A,B). Notably, the mode sizes of these nanoparticles appeared to be similar to that of EVs. Next, we assessed the growth and morphology of stem cells in these alternative media types. After a 48 h incubation period, we found that both iPSCs (Supplementary Figures S1C,D) and ESCs (Supplementary Figures S1F,G) grew differently in these three media types. In PS media, cells appeared less viable based on cell morphology and had much lower total cell counts as compared to regular SR media. In OM media, stem cells proliferated at a higher rate and some colonies displayed altered morphology. As for EVs, the greatest number of particles was recovered from SR CM, followed by OM and lastly PS media (Supplementary Figures S1E,H). As we were concerned with the cellular changes caused by the switch in media type, we decided to continue with SR media for subsequent comparisons between UC and LC protocol.

### LC Enabled Purification of Stem Cell EVs in a Reproducible Fashion

Since SR media does not contain serum, the detection of vesicles by NTA in the UC pellet of SR media led us to question the nature of these particles. We hypothesized that these “particles” were macromolecular structures of protein aggregates that formed during the high-speed centrifugation.

**FIGURE 1:**
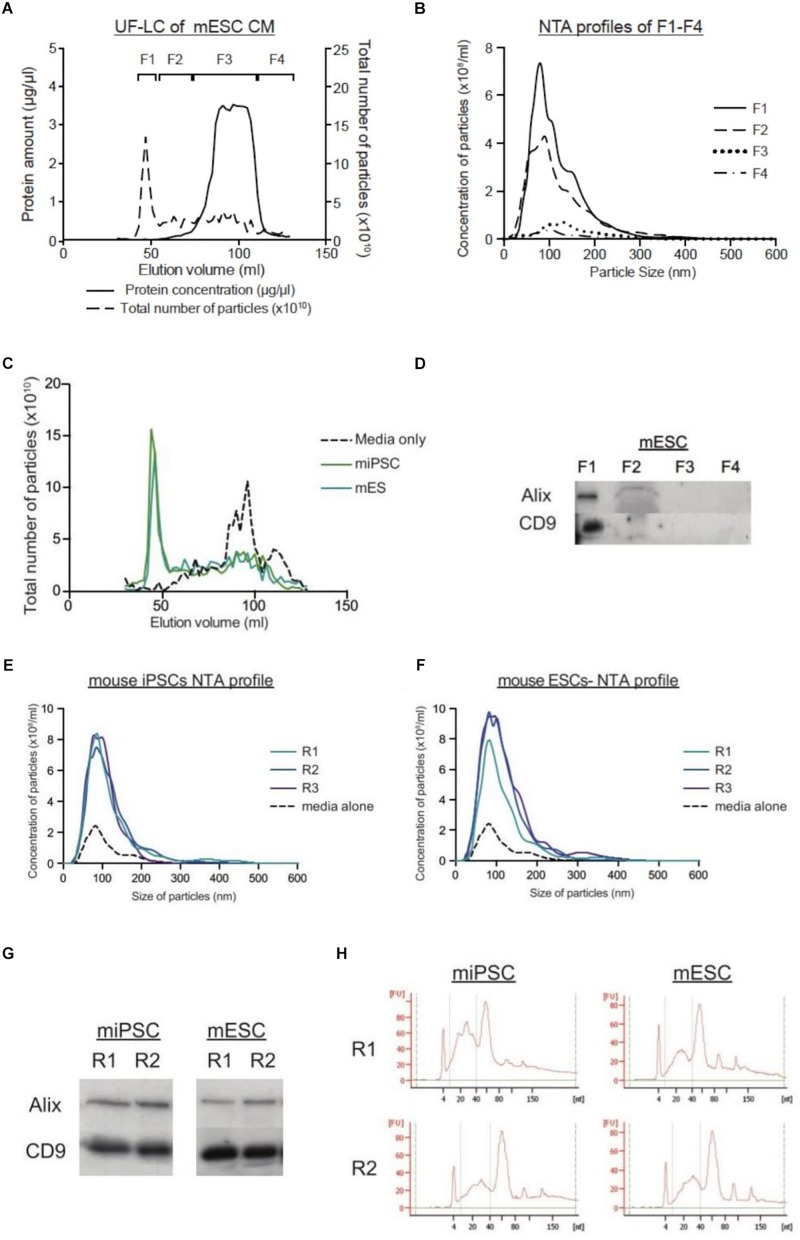
LC protocol allows for purification of EVs in a reproducible manner. **(A)** Graph showing the protein concentration (μg/μl) and the total number of particles (x10^10^) across the LC run. **(B)** NTA size distribution graphs of the four fractions from mESCs, where F1 is expected to contain the most EVs. **(C)** Graphs comparing the total number of particles (x10^10^) collected through the elution courses of unconditioned media, and CM from miPSCs and mESCs. **(D)** Representative western blots for EV markers (Alix and CD9) in mESC CM. NTA size distribution profiles of the EVs collected from LC-F1 in three replicates (R1–R3) of CM from miPSC **(E)** and mESC **(F)**. **(G)** Representative western blotting pictures for EV markers (Alix and CD9) in duplicate EVs samples (R1–R2) from miPSCs and mESCs. **(H)** Small RNA Bioanalyzer profiles of duplicate EV samples (R1–R2) from miPSCs and mESCs.

In LC, there is a lack of high-speed spins. Hence, we fractionated CM from stem cells on the size-exclusion column. Generally, the total protein and particle number profiles overlapped well, such that the fractions with the highest amounts of protein corresponded to the fractions with the highest number of particles (Figure 1A). Overall, we grouped and pooled the fractions into four general fractions: F1–F4. NTA profiles of these four fractions showed that the greatest number of particles to be in F1 followed by F2, while F3 and F4 contained very few particles (Figure 1B). When we compared their particle size distribution graphs, the peak of particle concentration in F1 was significantly higher in cell-derived samples than that of media alone (Figure 1C). Moreover, the presence of EVs exclusively in F1 was confirmed through the detection of common EV markers (Alix and CD9) (Figure 1D). Importantly, this showed that stem cell media contains a fair amount of macromolecular proteins that may appear as particles on NTA. Unlike in LC, these contaminating proteins are inseparable from EVs in the UC process. Furthermore, the LC process was highly reproducible across replicate runs, as seen by the protein chromatographs, western blotting and small RNA profile analysis of replicate collections (Figures 1E–H).

### UC Pelleting Led to Co-precipitation of Non-vesicular Proteins and RNAs With EVs

**FIGURE 2:**
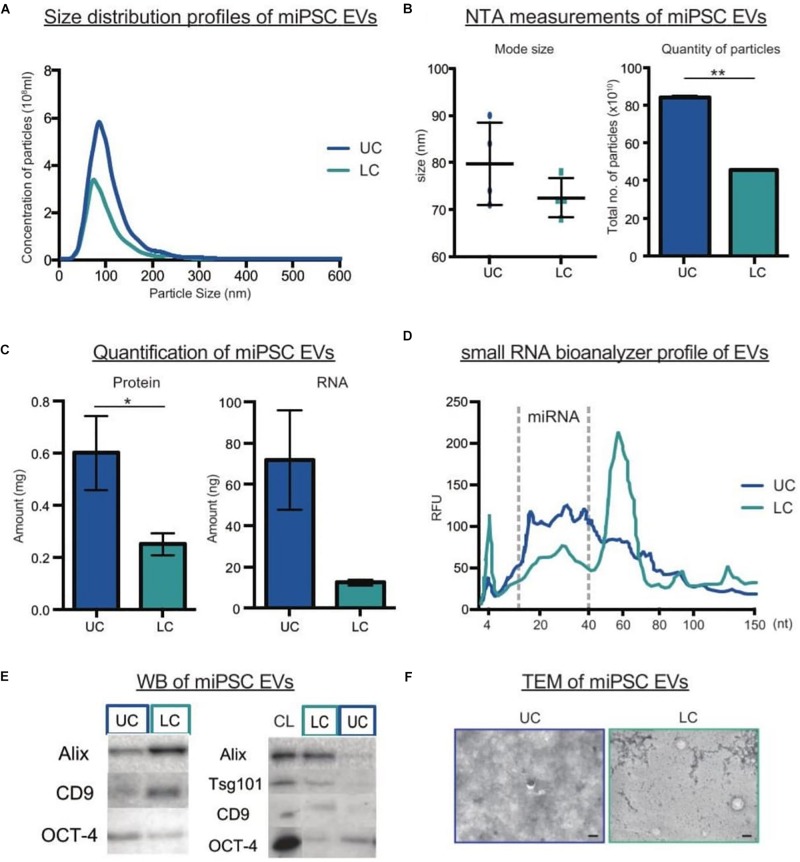
Comparison of miPSCs EVs purified by UC or LC. **(A)** NTA size distribution profiles of EVs purified by UC or LC. **(B)** The mode size (nm) and total number of particles (x10^10^) detected by NTA in UC or LC purified samples (*n* = 4, bars represent mean ± SD, ^∗∗^*p* < 0.01). **(C)** Protein and RNA quantification of EVs in UC and LC purified samples (*n* = 4, bars represent mean ± SD, ^∗^*p* < 0.05). **(D)** Small RNA Bioanalyzer profiles from UC and LC purified samples **(E)** Representative western blots of UC and LC purified samples. The left panel compares equal numbers of particles (4 × 10^10^), while the right panel compares equal amount of protein from (10 μg) of cell lysate (CL) and EV samples. **(F)** Representative TEM images of EVs purified by UC or LC. The scale bar corresponds to 100 nm.

When comparing UC and LC side-by-side, we detected higher particle yield and total protein and RNA amounts after UC as compared to LC (Figures 2A–D). However, this contradicted our previous study on serum-free culture samples (Nordin et al., 2015). To understand the reasons underlying the differences, we analyzed the expression levels of common EV markers following both types of collection. Interestingly, when normalized by particle numbers, we detected stronger expression of both Alix and CD9 in LC purified than UC purified samples, whereas OCT-4, a marker of pluripotency, showed the reverse trend (Figure 2E). Next, we compared the expression of the same EV markers in parental cells and EVs based on a fixed protein amount. Unexpectedly, there was no enrichment of EV markers in the UC pellet as compared to cells. To complement our molecular profiling, we next assessed how the biophysical properties of EVs differed by purification protocol. Overall, TEM imaging showed a general cloudy background in the UC samples, which made it difficult to discriminate EVs. In contrast, LC samples had a cleaner background where particles were more easily detected (Figure 2G). Lastly, we calculated the particle per protein ratio (P/μg) and compared them to benchmark values suggested for EV purity (Table 1). Here, the P/μg ratio for UC sample was classified as impure, while the LC samples had slightly higher P/μg ratios and were considered of low vesicular purity (Table 2).

**TABLE 1:** Table showing the suggested purity index by Webber and Clayton 2013.

**Purity index**	**P/μg**
High vesicular purity	>3 × 10^10^
Low vesicular purity	2 × 10^9^–2 × 10^10^
Unpure vesicles	1.5 × 10^9^

**TABLE 2:** Table showing the purity ratios of UC and LC samples from both stem cell types.

**P/μg**	**miPSC**	**mESC**
UC pellet	1.6 × 10^9^	4.5 × 10^8^
LC sample	3.6 × 10^9^	6.2 × 10^9^

**FIGURE 3:**
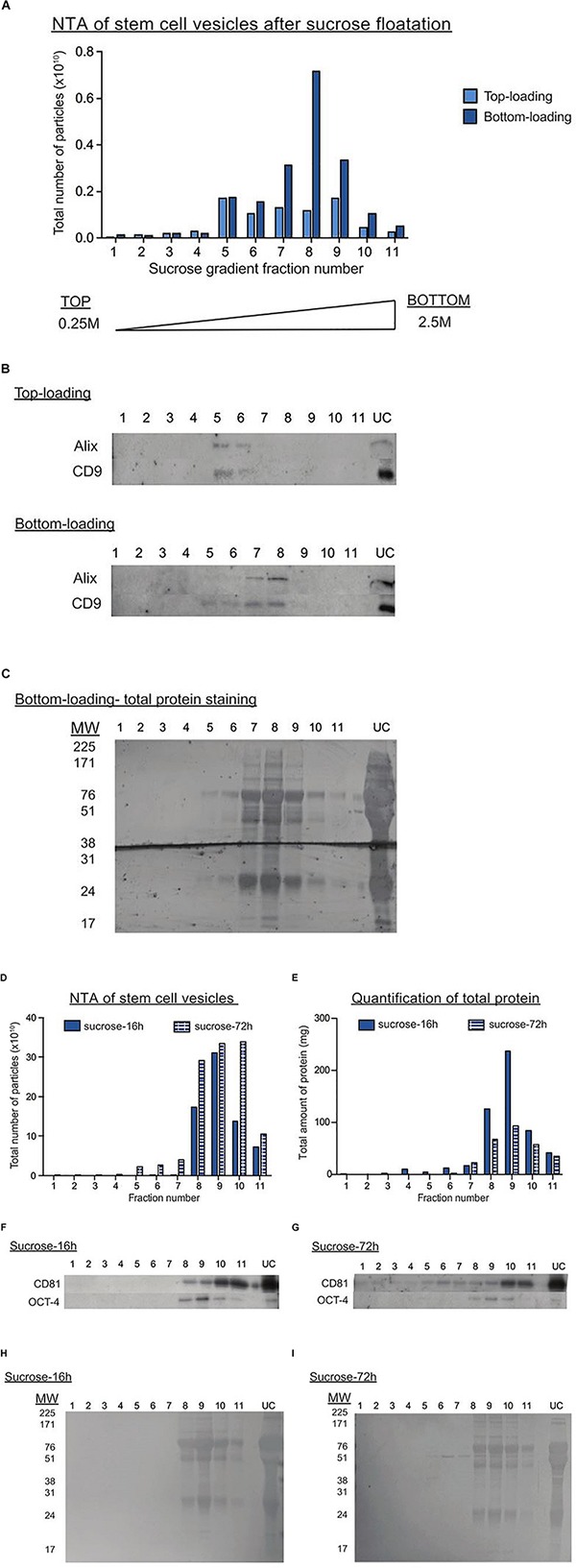
Sucrose gradient fractionation of UC pellets indicates the co-precipitation of non-vesicular proteins with EVs. **(A)** Graph showing the total number of particles detected in each sucrose gradient fraction after top (light blue) or bottom loading (dark blue) the sample. The fractions are numbered from the top to the bottom of the gradient. **(B)** Representative western blots for EV markers in the top and bottom loaded gradient fractions. UC represents the original UC pellet input material. **(C)** Total protein staining of gradient fractions from the bottom loaded sample. **(D,E)** Graphs showing the total number of particles **(D)** or protein **(E)** detected in each gradient fraction after UC samples were bottom-loaded and centrifuged for 16 h (dark blue bar) or 72 h (striped blue bars). Representative western blots for EV (CD81) and pluripotency (OCT4) markers in UC samples centrifuged on the sucrose gradient for either 16 h **(F)** or 72 h **(G)**. Total protein staining of gradient fractions from UC samples centrifuged on the sucrose gradient for either 16 h **(H)** or 72 h **(I)**.

One of the methods proposed for deriving pure, clinical-grade EVs is by floating EVs on a discontinuous sucrose gradient overnight (Lamparski et al., 2002), where pure EVs should float at a specific density range of 1.15–1.19 g/ml (Théry et al., 2006). Here, we compared EV purity after loading the sucrose gradient either above (bottom-loading) or below the UC pellet (top-loading). Interestingly, we found a discrepancy in the fractions enriched for EV markers and peak particle counts between the two loading types. After bottom-loading, the purified sample unexpectedly peaked in fractions 7–8, which correspond to a density of 1.21–1.22 g/ml (Figures 3A,B). These fractions also contained the greatest amounts of protein (Figure 3C). Hence, we speculated that non-vesicular proteins attached to EVs during the UC process and that this prevented the migration of EVs to their real densities. When further increasing the fractionation time in the sucrose gradient to 72 h, we began to detect more expression of the EV marker CD81 in the expected fractions of pure EVs (Figures 3D–I). Overall, we here show that initial quantification of particle counts and molecular content (proteins and RNAs) in UC pellets were inaccurate. Despite additional prolonged periods of clean-up by either sucrose gradient centrifugation or LC (Supplementary Figure S2), we achieved only a marginal improvement in the purity of EVs isolated by UC.

### EVs From iPSCs and ESCs Have Similar Size Distribution Profiles and Expression of EV Markers

**FIGURE 4:**
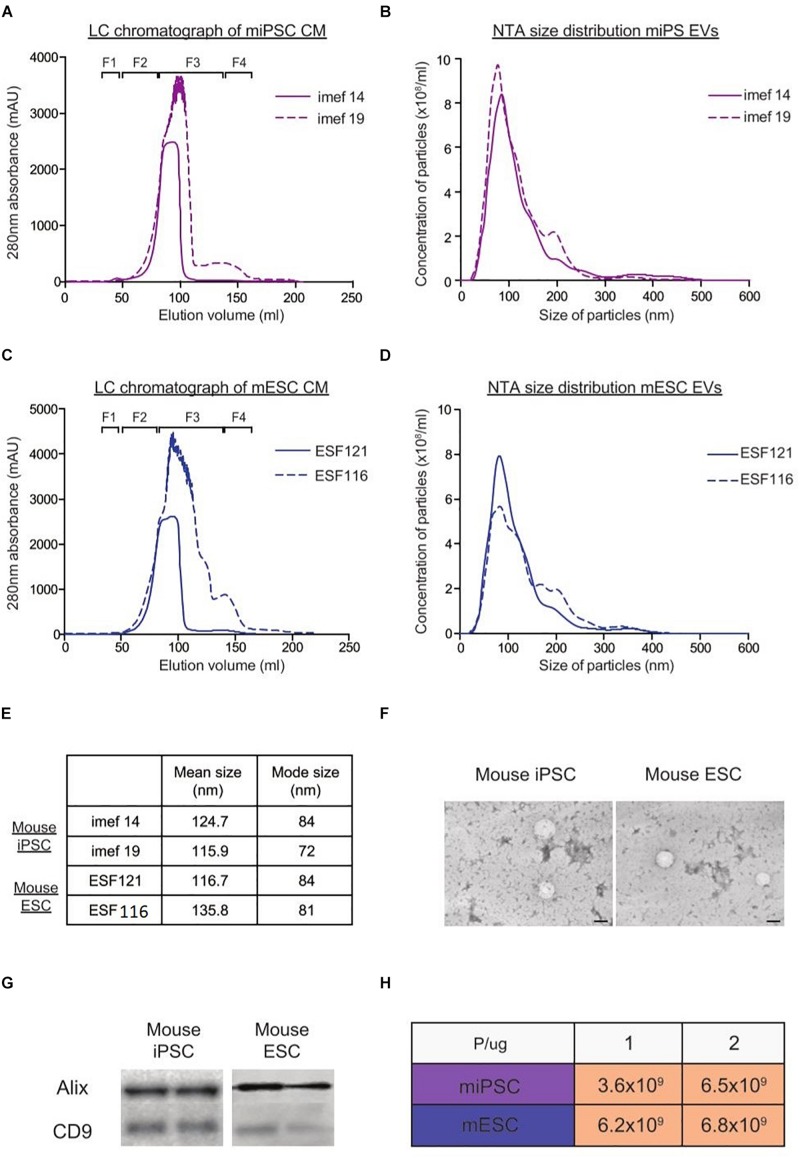
Characterization of EVs from mouse iPSCs and ESCs with NTA, TEM and western blotting. Representative LC chromatographs of two different mouse iPSC cell lines (imef14 and imef19) **(A)** and mouse ESC cell line (ESF121 and ESF116) **(B)**. NTA size distribution profiles of EVs from mouse iPSCs **(C)** and mouse ESCs **(D)**. **(E)** Table showing the mean and mode sizes of EVs from mouse iPSCs and ESCs. **(F)** Representative TEM images of EVs from mouse iPSCs and mouse ESCs. **(G)** Representative western blots of EV markers (Alix and CD9) in replicate samples of EVs from mouse iPSCs and mouse ESCs. **(H)** Table showing the P/μg ratio of EVs purified from mouse iPSCs and ESCs.

Using the LC method, we next proceeded to purify EVs from two mouse ESCs lines and two iPSCs lines. From the LC chromatograph, it was observed that all four EV fractionation processes generally showed a similar 280 nm absorbance pattern across the eluted volume (Figures 4A,C). As we were only interested in the EVs, we pooled LC fractions across the region where EVs eluted (F1) and analyzed the particles with NTA. As shown in Figures 4B,D,E, the particle size distribution profiles overlapped within each cell type. Moreover, TEM on EVs from both mouse ESCs and iPSCs corroborated the NTA measurements and showed that the particles from both cell types appeared similar. Furthermore, similar levels of EV markers (Alix and CD9) were detected in both mouse iPSC and ESC-derived EVs (Figures 4F,G). Lastly, when analyzing the P/μg ratio of EVs from all four cell lines, we found that all samples were of similar purity (Figure 4H).

### EVs From ESCs and iPSCs Contain Similar Proteins That Differ Greatly From Their Parental Cells

To characterize our EVs more deeply, unbiased LC-MS/MS proteomics analysis were performed on both cell lysates and EVs from our iPSC and ESC lines. As we suspected remnants of contaminating proteins within our EV samples, LC-MS/MS was first performed on unconditioned stem cell media. Overall, 35 cow and 29 mouse proteins were detected (Table 3), with the majority of proteins belonging to common protein groups such as albumin, fibronectin, heat shock protein and keratins (Tables 4–6). Importantly, many of these proteins were also detected in high abundance within our EV samples (Supplementary Figure S3). To avoid ambiguity, we removed the entire set of proteins present in media and our EV samples from downstream analysis.

**TABLE 3:** Table showing the list of cow proteins exclusively identified in the media sample.

**Species**	**Name of protein**	**Accession number**
**of protein**		
Bos taurus	Alpha-1-acid glycoprotein	Q3SZR3
	Alpha-1 -antiproteinase	P34955
	Alpha-1 B-glycoprotein	Q2KJF1
	Alpha-2-antiplasmin	P28800
	Alpha-2-HS-glycoprotein	P12763
	Alpha-2-macroglobulin	Q7SIH1
	Antithrombin-lll	F1MSZ6
	Apolipoprotein A-l	P15497
	Apolipoprotein A-IV	F1N3Q7
	C1QC protein (Fragment)	Q1RMH5
	Complement C3	G3×7A5
	Complement component C9	Q3MHN2
	FGG protein	Q3SZZ9
	Fibrinogen alpha chain	A5PJE3
	Fibrinogen beta chain	F1MAV0
	Gelsolin	F1MJH1
	Haptoglobin	G3 × 6K8
	Hemoglobin subunit beta	P02070
	Inter-alpha-trypsin inhibitor heavy chain H1	F1MMP5
	Inter-alpha-trypsin inhibitor heavy chain H4	F1MMD7
	Leucine-rich alpha-2-glycoprotein 1	Q2KIF2
	Protein AMBP	F1MMK9
	Prothrombin	P00735
	Serotransferrin	Q29443
	Serpin A3-5	A2I7N1
	Serpin A3-7	A2I7N3
	Uncharacterized protein (Fragment)	G3N0V0
	Uncharacterized protein (Fragment)	G5E513
	Uncharacterized protein	F1N514
	Uncharacterized protein	F1N076
	Uncharacterized protein	E1BH06
	Uncharacterized protein	F1MLW8
	Uncharacterized protein	F1MCF8
	Vitamin D-binding protein	F1N5M2

**TABLE 4:** Table showing the list of mouse proteins exclusively identified in the media sample.

**Species**	**Class of**	**Name of**	**Accession**
**of protein**	**protein**	**protein**	**number**
Mus musculus	Fibronectin	Fibronectin	A0A087WS56
		Fibronectin	B7ZNJ1
	Heat shock protein	Heat shock protein 75 kDa, mitochondrial	Q9CQN1
		Heat shock protein HSP 90-alpha	P07901
		Heat shock protein HSP 90-beta	P11499
	Keratin	Keratin, type I cytoskeletal 16	Q9Z2K1
		Keratin, type I cytoskeletal 17	Q9QWL7
		Keratin, type I cytoskeletal 18	P05784
		Keratin, type I cytoskeletal 19	P19001
		Keratin, type II cytoskeletal 2 epidermal	Q3TTY5
		Keratin, type II cytoskeletal 8	P11679
	L-lactate dehydrogenase	L-lactate dehydrogenase A chain	P06151
		L-lactate dehydrogenase B chain	P16125
	Others	Actin, cytoplasmic 1	P60710
		Annexin (Fragment)	B0V2N7
		Apolipoprotein B-100 (Fragment)	E9Q1Y3
		Apoptosis facilitator Bcl-2-like protein 14	Q9CPT0
		Beta-actin-like protein 2	Q8BFZ3
		Complement component C8 beta chain	Q8BH35
		E3 ubiquitin-protein ligase TRIP12	A0A087WNZ7
		Filamin-C	D3YW87
		Fructose-bisphosphate aldolase A	P05064
		Gelsolin	P13020
		Hemoglobin subunit alpha	P01942
		Histone H4	P62806
		Junction plakoglobin	Q02257
		Rho guanine nucleotide exchange factor 9 (Fragment)	S4R1J2
		Ubiquitin-associated domain-containing protein 2	Q8R1K1

**TABLE 5:** Table showing the list of mouse proteins exclusively identified in the gelatin sample.

**Species**	**Class of**	**Name of protein**	**Accession**
**of protein**	**protein**		**number**
Mus	Collagen	Collagen alpha -1 (I) chain	P11087
musculus		Collagen alpha-1 (II) chain	P28481
		Collagen alpha-1 (III) chain	P08121
		Collagen alpha-1 (V) chain	088207
		Collagen alpha-2 (l) chain	Q01149
	Others	Ig gamma-2A chain C region secreted form	P01864
		Ig heavy chain V region MOPC 47A	P01786
		Protein Ahnak	E9Q616
		Protein BC067074 (Fragment)	F6Z6Y0

*List excludes the detection of 8 uncharacterized sus scrofa proteins.*

**TABLE 6:** Table showing the list of proteins commonly identified in both the media and gelatin sample.

**Species**	**Class of**	**Name of protein**	**Accession**
**of protein**	**protein**		**number**
Bos taurus	Albumin	Serum albumin	P02769
Mus musculus	Keratin	Keratin, type II cytoskeletal 79	Q8VED5
		Keratin, type II cytoskeletal 75	Q8BGZ7
		Keratin, type II cytoskeletal 5	Q922U2
		Keratin, type II cytoskeletal 1	P04104
		Keratin, type I cytoskeletal 10	A2A513

*List excludes 2 uncharacterized sus scrofa proteins.*

**FIGURE 5:**
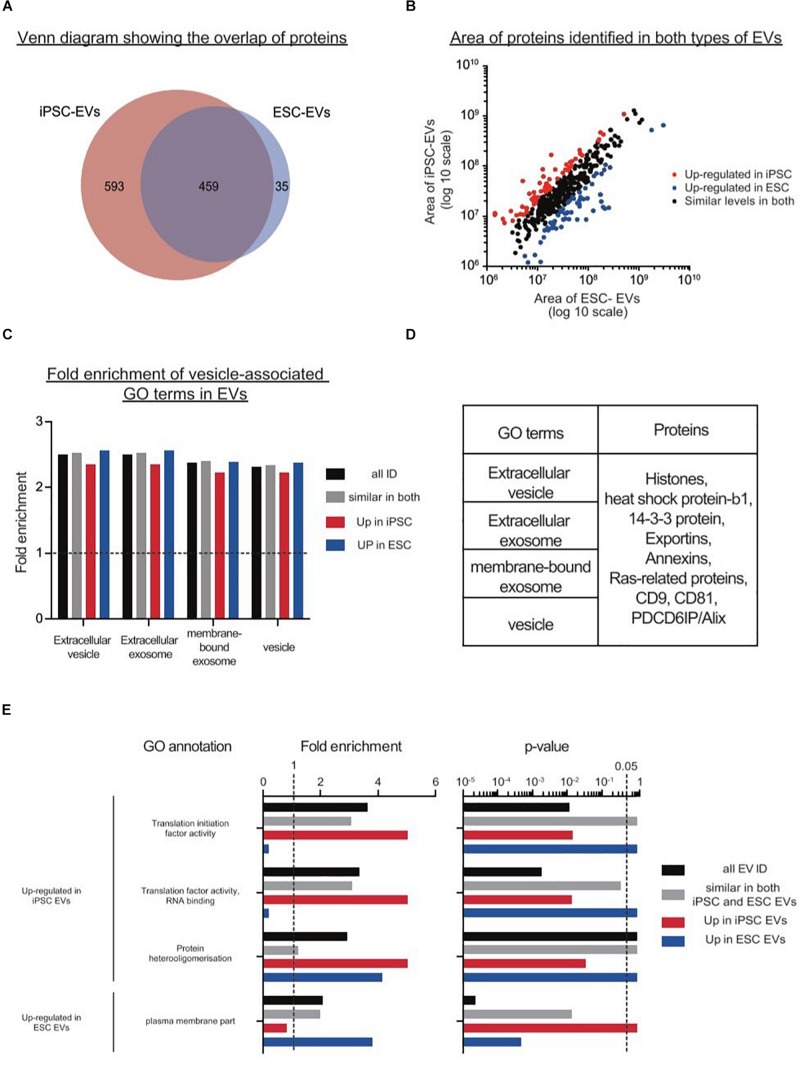
Proteomics analysis on EVs from mouse iPSCs and ESCs. **(A)** Venn diagram showing the overlap of proteins identified in the two types of stem cell EVs. **(B)** Scatter plot showing the correlation between the areas of commonly identified proteins in both iPSC-EVs and ESC-EVs. All proteins were classified into three distinct groups: up-regulated in iPSC-EVs (red dots), up-regulated in ESC-EVs (blue dots) and of similar levels in both iPSC and ESC-derived EVs (black dots). **(C)** Graph showing the fold enrichments of iPSC-EVs and ESC-EVs over the reference list, in four vesicle-related GO terms: extracellular vesicle, extracellular exosome, membrane-bound exosome and vesicle. **(D)** List of individual proteins identified in these same four GO terms. **(E)** A subset of significantly enriched GO terms for proteins up regulated in iPSC or ESC-derived EVs. Proteins are grouped under four categories: all EV IDs (black bar), proteins at similar levels in both iPSC-EVs and ESC-EVs (gray bar), up-regulated in iPSC-EVs (red bar) and up-regulated in ESC-EVs (blue bar).

After filtering, a total of 1387 proteins were detected in EVs from either iPSCs or ESCs, where 33.1% of them were present in both groups (Figure 5A). Although there were many more proteins found exclusively in iPSC-EVs than in ESC-EVs (593 vs. 35), GO annotations showed that many of these proteins had similar molecular functions, such as protein binding and cytosolic localization. Next, we considered the 459 proteins which were common to both types of EVs and divided them into three groups: proteins with similar expression, proteins over-represented in iPSC-EVs and proteins over-represented in ESC-EVs. In general, most of these common proteins were found at similar levels in both groups (Figure 5B). As expected, both EVs were enriched in proteins with GO terms such as EV, extracellular exosome, membrane-bound exosome and vesicle (Figure 5C). Moreover, known EV markers like CD9 and CD81 were abundant in both samples and were cross validated by western blotting (Figure 5D). On the other hand, a few GO terms were significantly different between the two groups. For example, GO terms for translation initiation factor activity, translation factor activity, RNA binding and protein hetero oligomerization were over-represented in EVs from iPSCs, while proteins associated with the plasma membrane were over-represented in EVs from ESCs (Figure 5E).

**FIGURE 6:**
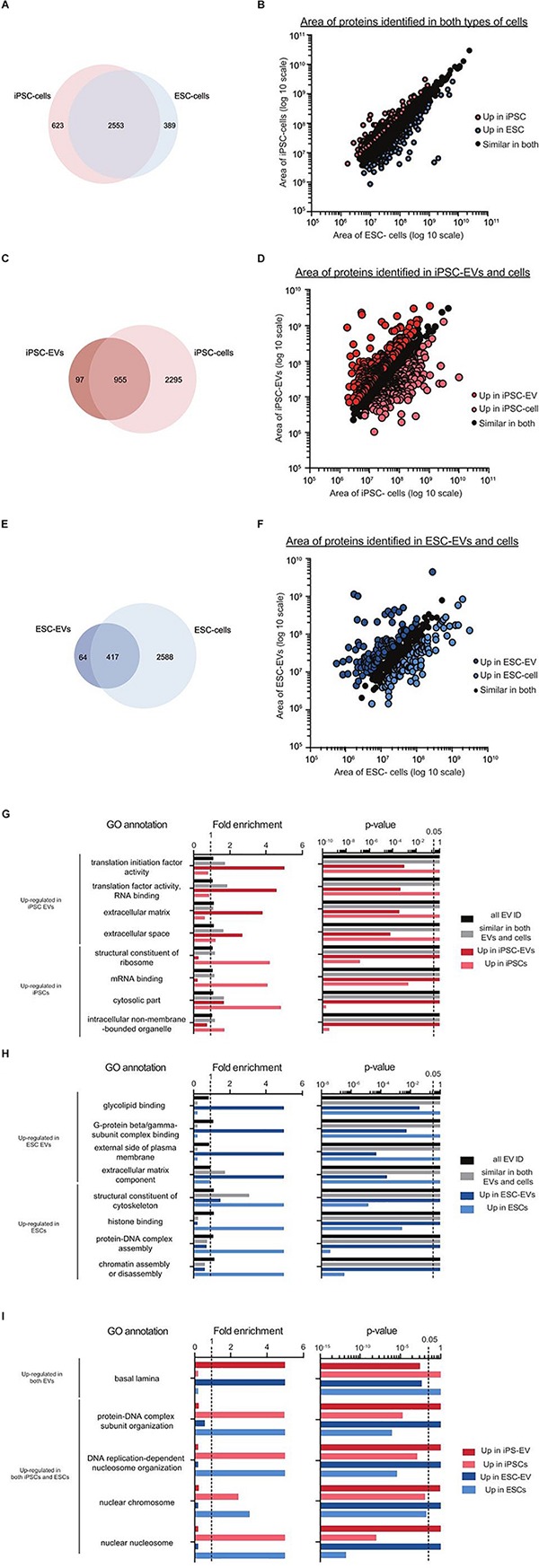
Preliminary proteomics comparing GO terms of proteins from mouse iPSC and ESC EVs and cells. Venn diagram showing the overlap of proteins identified in the two types of stem cells **(A)**, in mouse iPSC-EVs and iPSCs **(B)** and in mouse ESC-EVs and ESCs **(C)**. Scatter plots showing the correlation between the areas of commonly identified proteins in each of these three pairing: iPSCs versus ESCs **(D)**, iPSC-EVs versus iPSCs **(E)** and ESC-EVs versus ESCs **(F)**. All proteins were classified into three distinct groups as indicated in the graphs. A subset of significantly enriched GO terms for proteins considered to be up regulated in either iPSCs or ESCs **(G)**, iPSC-EVs or iPSCs **(H)**, and ESC-EVs or ESCs **(I)**.

To analyze if the similarities in the EVs was related to their parental cells, we next compared the cellular proteomes of matched iPSCs and ESCs to their respective EVs. As expected, more proteins were detected in cells than in EVs (3565 versus 1387). Of the proteins identified, 71.6% overlapped in both cell types (Figure 6A). As for proteins exclusive to each cell type, most of them mapped to the same GO terms (e.g., nucleotide and RNA binding, organelle and membrane). Similar to before, common proteins were separated into three groups; those expressed similarly in both, those over-represented in iPSCs and those over-represented in ESCs. Although there were variations in GO term enrichments between the two cell types, none of these differences were found to be significant. Interestingly, the scatter plot of individual proteins showed the exception of two outliers: mitochondrial ribosomal protein S25 (MRPS25) and activating signal co-integrator 1 complex subunit 3 (ASCC3), both of which were more abundant in ESCs than in iPSCs (Figure 6B).

Next, we compared each EV sample back to their parent cells. Interestingly, the overlap of cellular proteins in their corresponding EVs was quite low; 28.5% for iPSCs and 15.5% for ESCs (Figures 6C,E). This was further supported by the scatter plots showing correlation expression of all identified proteins in EVs versus cells (Figures 6D,F). To understand the nature of these differences, we applied GO analysis to the different groups of proteins. In the iPSC set, GO terms for translation initiation factor activity, translation factor activity, RNA binding, extracellular matrix and extracellular space were all found to be over-represented in EVs. In contrast, proteins with GO terms for structural constituent of ribosome, mRNA binding, cytosolic part and intracellular non-membrane-bounded organelle were over-represented in cells (Figure 6G). As proteins detected in ESC-EVs, we observed an enrichment in GO terms for glycolipid binding, G-protein beta/gamma-subunit complex binding, external side of plasma membrane and extracellular matrix component in the EVs. In contrast, ESC proteins were over-represented in proteins with GO terms for the structural constituent of cytoskeleton, histone binding, protein-DNA complex assembly and chromatin assembly or disassembly, as compared to EVs (Figure 6H). Interestingly, there were some GO terms which were similarly enriched in EVs and cells across both stem cell types. For example, the GO term basal lamina was enriched in EVs from both iPSCs and ESCs when compared to their parent cells. On the other hand, GO terms for protein-DNA complex subunit organization, DNA replication-dependent nucleosome organization, nuclear chromosome and nuclear nucleosome were enriched in both cell samples as compared to their EVs (Figure 6I).

## Discussion

In this study, we cross compared the isolation of EVs from complex, protein rich media using two methods: the gold-standard of UC versus an LC method. Due to the presence of microvesicles in serum, many researchers have turned to alternative media types like pre-spun media or entirely serum-free solutions reconstituted with recombinant protein substitutes for serum component (Li et al., 2015). Unfortunately, there have been doubts about the clearance efficiency of microvesicles from pre-spun media (Kornilov et al., 2018), and it is not feasible to reconstitute serum components for certain sensitive cell types. Here, we have demonstrated the phenotypic changes that occur in stem cells after short term incubation in these different media types. Although serum-free alternatives for cell types such as neurons and astrocytes have been developed (Brewer, 1995; Pozzi et al., 2017), these remain poorly defined, protein rich alternatives that may induce unwanted molecular changes and lead to growth of selective populations (Balvers et al., 2013). Despite the growing number of studies comparing EV isolation methods, there is still only limited knowledge about the molecular profiles of EVs collected from protein rich media types and their implications on molecular contents of EVs.

In our findings, we highlight various problems associated with the UC protocol. First, we identify an issue with high particle counts in UC pellets from unconditioned media, despite the lack of serum in KOSR. However, when we fractionated the unconditioned media with LC, most particles were found in later fractions corresponding with high protein amounts. Hence, this led us to speculate that the high-speed spins in UC promote the precipitation of protein aggregates, which then appear as nano-sized particles on NTA.

In our side-by-side comparison of UC and LC, the UC pellet contained more particles, protein and RNA than the LC sample. This result was rather unexpected, as the opposite trend was previously reported in serum-free CM (Nordin et al., 2015). As we are aware that protein aggregates in the UC pellet can appear as particles on the NTA, we remain unsure of the reliability of our NTA and protein quantifications. Thus, we used multitude of other technologies for our subsequent detailed comparison and characterization studies.

First, we checked the expression levels of well-established EV and stem cell markers when loading equal numbers of particles. Surprisingly, the expression level of OCT-4 was inversely correlated to that of the EV markers. Although OCT-4 is reported to be present in ESC-EVs (Ratajczak et al., 2006), more recent work has shown that OCT-4 can also be secreted from cells directly into the extracellular environment (Wang and Jauch, 2014). Hence, we speculate that some portion of this secreted OCT-4 protein in the CM might pellet together with stem cell EVs during the UC process. This hypothesis would explain the high particle counts and levels of OCT-4 detected by western blotting in UC-purified EVs. Apart from proteins, we also observed significant differences in other EV properties after isolation with UC and LC, such as their small RNA profiles and TEM images of vesicles. In the latter, we hypothesized that the presence of free protein in the UC pellet would contribute to the cloudy background that interfered with our visualization of the EVs. Furthermore, the P/μg ratio was lower after UC than LC purification; which corroborated our data suggesting that the UC pellet displayed lower vesicle purity. However, here we realize that interpretation of the vesicle purity ratio (P/μg) may need to be re-considered for protein rich substances. Since some protein aggregates may be mistakenly counted as EVs when using NTA, purified samples with more protein aggregates would appear to have a higher purity ratio than they do.

Next, we subjected the UC pellet to additional clean-up strategies to test for the presence of contaminating proteins. The first clean up strategy we attempted was sucrose gradient centrifugation. Previously, it has been reported that EVs float at 1.15–1.19 g/ml, which is slightly different to vesicles originating from cellular organelles such as the endoplasmic reticulum (1.18–1.25 g/ml) or the Golgi (1.05–1.12 g/ml) (Théry et al., 2006). These floatation intervals have been verified on EVs from different cellular sources and found to be reproducible regardless of the method of loading the EV sample on the top or bottom of the sucrose gradient. However, others have debated that overnight centrifugation was insufficient for vesicles to efficiently penetrate in the denser fractions and reach density equilibrium within 16 h centrifugation period (Yuana et al., 2014; Iwai et al., 2016). Hence, we here tested both loading strategies (top versus bottom) and at two different lengths of centrifugation timings (16 h versus 72 h). Unexpectedly, there was great inconsistency in the fractions showing the highest total particle counts and expression of EV markers between the two loading strategies. In the bottom-loaded sample, the gradient fractions positive for EV markers overlapped with those containing non-vesicular protein bands. Hence, we hypothesize that the EVs in the UC pellet may be associated with protein aggregates and thus float at these higher densities instead of their actual density.

Interestingly, with the longer overnight centrifugation, the expression of CD81 became more diverse. We hypothesized that after this longer incubation period, a portion of EVs were able to escape from the protein aggregates and float at their expected densities. In hindsight though, CD81 expression still appeared relatively higher in the higher density fractions. Despite this data, it is very difficult to conclude whether the particles appearing at higher densities and stained positively for CD81 are EVs bound to non-vesicular proteins or they could be true EVs that float at a higher density. However, there is the possibility that some of these EV-protein associations are transient, whilst others may be more permanent due the high gravitational forces inherent in the UC procedure. Another strategy we tried was to fractionate the UC pellet on an LC column. Interestingly, this revealed an additional protein peak immediately after the initial EV peak, though the particles in this second peak did not express any of the EV markers we tested. This result further supports our previous deduction that proteins can appear as particles on the NTA. From these results, we conclude that UC purification of EVs may require additional clean-up to increase the purity of EVs, despite that these steps would compromise the final EV yield as seen by another recent study (Onódi et al., 2018).

When applying LC to stem cell CM, the fractionation pattern was reproducible across replicates and consistent across the two stem cell types. However, we noticed that there was a lack of distinct cut-off points between the particle and protein peaks in the LC profile of stem cell CM. This led us to speculate that, due to the high protein content in the media, the resolution of fractionation may require more improvements; this can be in the form of sequential LC steps or with an alternative taller column. We briefly tested the sequential LC approach here and noticed that in the second LC run there was slightly lower expression of the transmembrane protein CD9, while the opposite was true for the intraluminal protein (Tsg101) (Supplementary Figure S4). Interestingly, this suggests that more caution is required when checking the expression of established EV markers identification. In this scenario, the lower expression of CD9 in the second LC may indicate that some CD9 initially detected might be contributed by non-EV proteins.

Despite our efforts to clean-up the EV product, there remains the presence of huge protein bands as seen by Ponceau S staining. From literature, KOSR contains high amounts of albumin and other proteins to replace the serum component. We postulated that the pre-concentration step with spin filters might have caused the capture of these protein aggregates in the filters. To bypass this, one could consider the use of tangential flow filtration (TFF) devices, which function through diafiltration-based processes. On the other hand, some contaminating proteins on EVs may be due to the presence of cell adhesion molecules like connexins, integrins and cadherins (Albelda and Buck, 1990; Sakisaka, 2005; Shimaoka et al., 2019) which would make EVs particularly adhesive to free proteins in the circulation.

Unlike previous studies on stem cell derived-EVs, which were collected from serum-free conditions (Ratajczak et al., 2006; Yuan et al., 2009; Katsman et al., 2012), we used serum-replacement media to avoid the differentiation of our stem cells in the absence of serum. To identify the background of proteins in the unconditioned media, we applied the use of a mass spectrometry method. We found a relatively high amount of cow albumin and keratin proteins. As the serum-replacement media contained AlbuMax^TM^ (Life Technologies, United Kingdom), we believed that this was the main source of cow albumin in our samples and pre-filtered these proteins out of downstream analysis.

Generally, the EVs from iPSCs and ESCs have relatively similar proteomes. Although there was a disparity in the total number of proteins detected in the iPSC-EVs and ESC-EVs, most of the specifically expressed proteins did not show a significant difference in GO terms enrichments and may not impact on cell functionality. Furthermore, our proteomics data highlighted the identities of other EV markers, which could serve as useful indicators for future studies on stem cell EVs.

Next, to trace if the similarities between EVs was related to their parental cells, we analyzed the proteomes of the cells from which the EVs were derived from. Although the proteomes of both stem cell types were highly similar, there was much less overlap between EVs and their corresponding parental source. This was largely due to the lower overall number of detected proteins in EVs than in cells. This is not at all unexpected, as it can be attributed to the size differences between a vesicle (100 nm) and a cell (>5 μm). However, the enrichment of specific GO terms in EVs over cells illustrates the differences between their biological contents and may be linked to their downstream functionality. In hindsight though, we cannot be certain how the background from the basal media may have also affected the LC-MS run and the downstream protein readout.

There is an overwhelming amount of research commenting on the similarities and differences between ESCs and iPSCs (Boulting et al., 2011; Narsinh et al., 2011; Phanstiel et al., 2011), and the variation between ESC and iPSC clones due to the type of donor cells (Ward et al., 2004; Osafune et al., 2008; Narsinh et al., 2011), their methylation status and transcriptomes (Kim et al., 2011; Lister et al., 2011; Ohi et al., 2011).

On the other hand, There have only been a handful of published proteomics studies on mouse (Graumann et al., 2007) and human iPSCs and ESCs (Phanstiel et al., 2011; Munoz et al., 2014), all of which have been performed on different ESC and iPSC lines. There have also been some recent studies on EVs from iPSCs and iPSC-derivatives (Zhou et al., 2016; Liu et al., 2017; Zhu et al., 2017; Ding et al., 2018; Dougherty et al., 2018; La Greca et al., 2018; Oh et al., 2018), though there is yet to be any proteomic data on EVs from pluripotent cell sources. Hence, due to the variation between lines, the biological data here might not be applicable to all other pluripotent stem cells.

In neurological diseases, standard methods of monitoring such as CT or MRI scans are costly, less sensitive and requires expertise interpretation. The use of traditional tissue biopsy or blood liquid biopsy in CNS-related disease has been elusive due to the presence of the blood brain barrier functions (Daneman and Prat, 2015). Recently though, some have capitalized on EVs in the cerebrospinal fluid (CSF) to monitor the brain and CNS (Welton et al., 2017; Thompson et al., 2018; Miller et al., 2019; Otake et al., 2019). However, the lumbar tap procedure for CSF extraction is painful and often only very small volumes of CSF can be extracted. Hence, we envision here that our study will assist other EV researchers on the methodology for EV purification from these precious sources.

Besides biology, EVs can also be exploited by loading therapeutic cargoes; both naturally and after engineering. In the former, there are many excellent reviews on how EVs from mesenchymal stem cells (MSCs) can alleviate disease through EV therapy in CNS-related diseases (Kawikova and Askenase, 2015; Koniusz et al., 2016; Luarte et al., 2016). On the other hand, others have cleverly designed targeting moieties on the surface of EVs and encapsulated genetic cargoes or drugs for therapeutic purposes (Alvarez-Erviti et al., 2011; El Andaloussi et al., 2013; Kalani et al., 2016; Usman et al., 2018). Here, we describe EVs from iPSCs that may serve as an alternative resource. Furthermore, we performed unbiased protein characterization of these EVs with the hope that this can assist in the future design and molecular engineering of EVs for specific personalized therapy.

## Conclusion

Here, we describe a study investigating the isolation of EVs from protein-rich complex media types, using stem cells as a model. Initially, our step-by-step comparison between UC and LC contradicted our expectations. Using additional technologies, we illustrate the possible causes and sources of protein contamination in the UC pellet and how they can be reduced with additional clean-up steps. However, these processes are somewhat inefficient and compromise on the final output. On the other hand, although LC was a better alternative to UC, the resultant EV population still appears to be of low purity. The purity of EVs is of great importance in all forms of EV research, from deducing biological functions to use as therapeutic agents, as contaminations may confound interpretations. Furthermore, we highlight the need to be cautious when using current tools for the interpretation of EVs purified from complex media or biological fluids. Lastly, we analyzed the protein content of these EVs and briefly discuss their potential in future CNS-related research.

## Data Availability Statement

All datasets generated for this study are included in the manuscript/Supplementary Files.

## Author Contributions

YL performed all the experiments and wrote the manuscript. HJ performed the proteomics. MW and SE conceptualized the study and provided critical discussions on the overall study and manuscript.

## Conflict of Interest

MW and SE are founders and shareholders of Evox Therapeutics. The remaining authors declare that the research was conducted in the absence of any commercial or financial relationships that could be construed as a potential conflict of interest.

## Supplementary Material

FIGURE S1 Alternative types of serum-depleted media for EV collection from stem cells. **(A)** NTA size distribution profiles of UC pellets after centrifugation of the three types of media: KOSR-supplemented stem cell media (SR), Pre-spun stem cell media (PS) and serum-free media/OptiMEM (OM). **(B)** Table showing the mode size (nm) and concentration of particles (x10^9^/ml) detected in the UC pellet of each media type. Representative bright-field images of mouse iPSCs **(C)** and mouse ESCs **(F)** after 48 h culture in SR, PS, or OM. Graphs comparing the total number of viable cells in the miPSCs **(D)** and mESCs **(E)** cultures at CM collection. Graphs comparing the total concentration of particles (x10^10^/ml) detected by NTA in the UC pellet of miPSCs **(G)** and mESCs **(H)** in the three different media types.

FIGURE S2 LC fractionation of UC pellets reveals co-precipitation of non-vesicular proteins with EVs in the UC pellet. **(A)** Schematic outline of the LC fractionation protocol of UC pellets. **(B)** LC chromatograph showing the 280 nm absorbance of the elution course from the LC column for three replicate samples (R1–R3). The first fraction (pink box) corresponds to the region where EVs elute. The second fraction (orange box) shows the appearance of another peak after the EVs. **(C)** NTA size distribution profiles of particles in the original UC sample (blue), and in the first (box 1; UC-LC1) and second (box 2; UC-LC2) LC fractions. **(D)** Graph on the left shows the mode size (nm) of particles in the original UC pellet, UC-LC1 and UC-LC2 (*n* = 3, bar represents mean ± SD). Graph on the right shows the percentage of particles detected in the UC pellet versus UC-LC1 (*n* = 3, bar represents mean ± SD). **(E)** Representative western blots for EV (Alix and CD9) and pluripotency (OCT4) markers when loading the same amount of particles from the UC pellet and UC-LC1 fraction. **(F)** Total protein staining of the UC pellet, UC-LC1 and UC-LC2. **(G)** Representative TEM images of the UC pellet and UC-LC1. Here the UC-LC1 sample appears to have a decreased background as compared to UC. The scale bar corresponds to 100 nm.

FIGURE S3 Detection of non-mouse proteins in mouse derived EVs. Scatter plot showing the areas of proteins identified in the preliminary analysis of iPSC- and ESC-EV samples. Proteins from mouse (black dots), cow (red dots) and pig (blue dots) are indicated.

FIGURE S4 Sequential LC fraction improves EV purity marginally. **(A)** Schematic outline of the sequential LC fractionation set-up. **(B)** LC chromatograph showing the 280 nm absorbance of the elution course from the LC column for three replicate samples (R1–R3). The first fraction (green box) corresponds to the region where EVs elute. **(C)** NTA size distribution profiles of particles in the first LC sample versus the second LC run (LC′1). **(D)** On the left, the mode size of particles in the original LC sample and the LC′1 sample appears to be similar (*n* = 3). On the right, graph showing overall percentage of particles recovered as compared to the input material. **(E)** Representative western blotting pictures when loading the same amount of particles for LC and LC’1. **(F)** Total protein staining of the membrane with LC and LC’1 samples.

TABLE S1 Table showing the protein expression of replicate runs of cells and EVs purified from mouse ESC and mouse iPSC.

## References

[B1] Albelda S. M. Buck C. A. (1990). Integrins and other cell adhesion molecules. *FASEB J.* 4 2868–2880. 10.1096/fasebj.4.11.2199285 2199285

[B2] Alvarez M. L. Khosroheidari M. Kanchi Ravi R. DiStefano J. K. (2012). Comparison of protein, microRNA, and mRNA yields using different methods of urinary exosome isolation for the discovery of kidney disease biomarkers. *Kidney Int.* 82 1024–1032. 10.1038/ki.2012.256 22785172

[B3] Alvarez-Erviti L. Seow Y. Yin H. Betts C. Lakhal S. Wood M. J. A. (2011). Delivery of siRNA to the mouse brain by systemic injection of targeted exosomes. *Nat. Biotechnol.* 29 341–345. 10.1038/nbt.1807 21423189

[B4] Antonucci F. Turola E. Riganti L. Caleo M. Gabrielli M. Perrotta C. (2012). Microvesicles released from microglia stimulate synaptic activity via enhanced sphingolipid metabolism. *EMBO J.* 31 1231–1240. 10.1038/emboj.2011.489 22246184 PMC3297996

[B5] Arslan F. Lai R. C. Smeets M. B. Akeroyd L. Choo A. Aguor E. N. E. (2013). Mesenchymal stem cell-derived exosomes increase ATP levels, decrease oxidative stress and activate PI3K/Akt pathway to enhance myocardial viability and prevent adverse remodeling after myocardial ischemia/reperfusion injury. *Stem Cell Res.* 10 301–312. 10.1016/j.scr.2013.01.002 23399448

[B6] Bahi A. Dreyer J.-L. (2005). Cocaine-induced expression changes of axon guidance molecules in the adult rat brain. *Mol. Cell. Neurosci.* 28 275–291. 10.1016/j.mcn.2004.09.011 15691709

[B7] Balvers R. K. Kleijn A. Kloezeman J. J. French P. J. Kremer A. van den Bent M. J. (2013). Serum-free culture success of glial tumors is related to specific molecular profiles and expression of extracellular matrix-associated gene modules. *Neuro Oncol.* 15 1684–1695. 10.1093/neuonc/not116 24046260 PMC3829587

[B8] Bobrie A. Colombo M. Krumeich S. Raposo G. Théry C. (2012). Diverse subpopulations of vesicles secreted by different intracellular mechanisms are present in exosome preparations obtained by differential ultracentrifugation. *J. Extracell. Vesicles* 1:18397. 10.3402/jev.v1i0.18397 24009879 PMC3760636

[B9] Böing A. N. van der Pol E. Grootemaat A. E. Coumans F. A. W. Sturk A. Nieuwland R. (2014). Single-step isolation of extracellular vesicles by size-exclusion chromatography. *J. Extracell. Vesicles* 3:23430. 10.3402/jev.v3.23430 25279113 PMC4159761

[B10] Boulting G. L. Kiskinis E. Croft G. F. Amoroso M. W. Oakley D. H. Wainger B. J. (2011). A functionally characterized test set of human induced pluripotent stem cells. *Nat. Biotechnol.* 29 279–286. 10.1038/nbt.1783 21293464 PMC3229307

[B11] Brewer G. J. (1995). Serum-free B27/neurobasal medium supports differentiated growth of neurons from the striatum, substantia nigra, septum, cerebral cortex, cerebellum, and dentate gyrus. *J. Neurosci. Res.* 42 674–683. 10.1002/jnr.490420510 8600300

[B12] Chen T. S. Arslan F. Yin Y. Tan S. S. Lai R. C. Choo A. B. H. (2011). Enabling a robust scalable manufacturing process for therapeutic exosomes through oncogenic immortalization of human ESC-derived MSCs. *J. Transl. Med.* 9:47. 10.1186/1479-5876-9-47 21513579 PMC3100248

[B13] Cvjetkovic A. Lötvall J. Lässer C. (2014). The influence of rotor type and centrifugation time on the yield and purity of extracellular vesicles. *J. Extracell. Vesicles* 3:23111. 10.3402/jev.v3.23111 24678386 PMC3967015

[B14] Daneman R. Prat A. (2015). The blood–brain barrier. *Cold Spring Harb. Perspect. Biol.* 7:a020412. 10.1101/cshperspect.a020412 25561720 PMC4292164

[B15] Ding Q. Sun R. Wang P. Zhang H. Xiang M. Meng D. (2018). Protective effects of human induced pluripotent stem cell-derived exosomes on high glucose-induced injury in human endothelial cells. *Exp. Ther. Med.* 15 4791–4797. 10.3892/etm.2018.6059 29805497 PMC5958753

[B16] Dougherty J. A. Kumar N. Noor M. Angelos M. G. Khan M. Chen C.-A. (2018). Extracellular vesicles released by human induced-pluripotent stem cell-derived cardiomyocytes promote angiogenesis. *Front. Physiol.* 9:1794. 10.3389/fphys.2018.01794 30618806 PMC6302004

[B17] El Andaloussi S. Mäger I. Breakefield X. O. Wood M. J. A. (2013). Extracellular vesicles: biology and emerging therapeutic opportunities. *Nat. Rev. Drug Discov.* 12 347–357. 10.1038/nrd3978 23584393

[B18] Fairchild P. J. Brook F. A. Gardner R. L. Graça L. Strong V. Tone Y. (2000). Directed differentiation of dendritic cells from mouse embryonic stem cells. *Curr. Biol.* 10 1515–1518. 10.1016/s0960-9822(00)00824-821 11114519

[B19] Fauré J. Lachenal G. Court M. Hirrlinger J. Chatellard-Causse C. Blot B. (2006). Exosomes are released by cultured cortical neurones. *Mol. Cell. Neurosci.* 31 642–648. 10.1016/j.mcn.2005.12.003 16446100

[B20] Frühbeis C. Fröhlich D. Kuo W. P. Amphornrat J. Thilemann S. Saab A. S. (2013). Neurotransmitter-triggered transfer of exosomes mediates oligodendrocyte-neuron communication. *PLoS Biol.* 11:e1001604. 10.1371/journal.pbio.1001604 23874151 PMC3706306

[B21] Ghosh A. Davey M. Chute I. C. Griffiths S. G. Lewis S. Chacko S. (2014). Rapid isolation of extracellular vesicles from cell culture and biological fluids using a synthetic peptide with specific affinity for heat shock proteins. *PLoS One* 9:e110443. 10.1371/journal.pone.0110443 25329303 PMC4201556

[B22] Gould S. J. Raposo G. (2013). As we wait: coping with an imperfect nomenclature for extracellular vesicles. *J. Extracell. Vesicles* 2:20389. 10.3402/jev.v2i0.20389 24009890 PMC3760635

[B23] Graumann J. Hubner N. C. Kim J. B. Ko K. Moser M. Kumar C. (2007). Stable isotope labeling by amino acids in cell culture (SILAC) and proteome quantitation of mouse embryonic stem cells to a depth of 5,111 proteins. *Mol. Cell. Proteomics* 7 672–683. 10.1074/mcp.M700460-MCP200 18045802

[B24] Harding C. Heuser J. Stahl P. (1984). Endocytosis and intracellular processing of transferrin and colloidal gold-transferrin in rat reticulocytes: demonstration of a pathway for receptor shedding. *Eur. J. Cell Biol.* 35 256–263. 6151502

[B25] Hienola A. Tumova S. Kulesskiy E. Rauvala H. (2006). N-syndecan deficiency impairs neural migration in brain. *J. Cell Biol.* 174 569–580. 10.1083/jcb.200602043 16908672 PMC2064262

[B26] Hoshino A. Costa-Silva B. Shen T.-L. Rodrigues G. Hashimoto A. Tesic Mark M. (2015). Tumour exosome integrins determine organotropic metastasis. *Nature* 527 329–335. 10.1038/nature15756 26524530 PMC4788391

[B27] Hung M. E. Leonard J. N. (2016). A platform for actively loading cargo RNA to elucidate limiting steps in EV-mediated delivery. *J. Extracell. Vesicles* 5:31027. 10.3402/jev.v5.31027 27189348 PMC4870355

[B28] Iwai K. Minamisawa T. Suga K. Yajima Y. Shiba K. (2016). Isolation of human salivary extracellular vesicles by iodixanol density gradient ultracentrifugation and their characterizations. *J. Extracell. Vesicles* 5:30829. 10.3402/jev.v5.30829 27193612 PMC4871899

[B29] Janas T. Janas M. M. Sapoń K. Janas T. (2015). Mechanisms of RNA loading into exosomes. *FEBS Lett.* 589 1391–1398. 10.1016/j.febslet.2015.04.036 25937124

[B30] Janowska-Wieczorek A. Wysoczynski M. Kijowski J. Marquez-Curtis L. Machalinski B. Ratajczak J. (2005). Microvesicles derived from activated platelets induce metastasis and angiogenesis in lung cancer. *Int. J. Cancer* 113 752–760. 10.1002/ijc.20657 15499615

[B31] Kalani A. Chaturvedi P. Kamat P. K. Maldonado C. Bauer P. Joshua I. G. (2016). Curcumin-loaded embryonic stem cell exosomes restored neurovascular unit following ischemia-reperfusion injury. *Int. J. Biochem. Cell Biol.* 79 360–369. 10.1016/j.biocel.2016.09.002 27594413 PMC5067233

[B32] Kanwar S. S. Dunlay C. J. Simeone D. M. Nagrath S. (2014). Microfluidic device (ExoChip) for on-chip isolation, quantification and characterization of circulating exosomes. *Lab. Chip* 14 1891–1900. 10.1039/c4lc00136b 24722878 PMC4134440

[B33] Katsman D. Stackpole E. J. Domin D. R. Farber D. B. (2012). Embryonic stem cell-derived microvesicles induce gene expression changes in Müller cells of the retina. *PLoS One* 7:e50417. 10.1371/journal.pone.0050417 23226281 PMC3511553

[B34] Kawikova I. Askenase P. W. (2015). Diagnostic and therapeutic potentials of exosomes in CNS diseases. *Brain Res.* 1617 63–71. 10.1016/j.brainres.2014.09.070 25304360 PMC4862949

[B35] Kim K. Zhao R. Doi A. Ng K. Unternaehrer J. Cahan P. (2011). Donor cell type can influence the epigenome and differentiation potential of human induced pluripotent stem cells. *Nat. Biotechnol.* 29 1117–1119. 10.1038/nbt.2052 22119740 PMC3357310

[B36] Koniusz S. Andrzejewska A. Muraca M. Srivastava A. K. Janowski M. Lukomska B. (2016). Extracellular vesicles in physiology, pathology, and therapy of the immune and central nervous system, with focus on extracellular vesicles derived from mesenchymal stem cells as therapeutic tools. *Front. Cell. Neurosci.* 10:109. 10.3389/fncel.2016.00109 27199663 PMC4852177

[B37] Kornilov R. Puhka M. Mannerström B. Hiidenmaa H. Peltoniemi H. Siljander P. (2018). Efficient ultrafiltration-based protocol to deplete extracellular vesicles from fetal bovine serum. *J. Extracell. Vesicles* 7:1422674. 10.1080/20013078.2017.1422674 29410778 PMC5795649

[B38] Krämer-Albers E.-M. Bretz N. Tenzer S. Winterstein C. Möbius W. Berger H. (2007). Oligodendrocytes secrete exosomes containing major myelin and stress-protective proteins: trophic support for axons? *Proteomics Clin. Appl.* 1 1446–1461. 10.1002/prca.200700522 21136642

[B39] La Greca A. Solari C. Furmento V. Lombardi A. Biani M. C. Aban C. (2018). Extracellular vesicles from pluripotent stem cell-derived mesenchymal stem cells acquire a stromal modulatory proteomic pattern during differentiation. *Exp. Mol. Med.* 50:119. 10.1038/s12276-018-0142-x 30201949 PMC6131549

[B40] Lachenal G. Pernet-Gallay K. Chivet M. Hemming F. J. Belly A. Bodon G. (2011). Release of exosomes from differentiated neurons and its regulation by synaptic glutamatergic activity. *Mol. Cell. Neurosci.* 46 409–418. 10.1016/j.mcn.2010.11.004 21111824

[B41] Lamparski H. G. Metha-Damani A. Yao J.-Y. Patel S. Hsu D.-H. Ruegg C. (2002). Production and characterization of clinical grade exosomes derived from dendritic cells. *J. Immunol. Methods* 270 211–226. 10.1016/s0022-1759(02)00330-7 12379326

[B42] Lane R. E. Korbie D. Trau M. Hill M. M. (2019). Optimizing size exclusion chromatography for extracellular vesicle enrichment and proteomic analysis from clinically relevant samples. *Proteomics* 19:e1800156. 10.1002/pmic.201800156 30632691

[B43] Lehrich B. Liang Y. Khosravi P. Federoff H. Fiandaca M. (2018). Fetal bovine serum-derived extracellular vesicles persist within vesicle-depleted culture media. *Int. J. Mol. Sci.* 19:3538. 10.3390/ijms19113538 30423996 PMC6275013

[B44] Li J. Lee Y. Johansson H. J. Mäger I. Vader P. Nordin J. Z. (2015). Serum-free culture alters the quantity and protein composition of neuroblastoma-derived extracellular vesicles. *J. Extracell. Vesicles* 4:26883. 10.3402/jev.v4.26883 26022510 PMC4447833

[B45] Lim J. Choi M. Lee H. Kim Y.-H. Han J.-Y. Lee E. S. (2019). Direct isolation and characterization of circulating exosomes from biological samples using magnetic nanowires. *J. Nanobiotechnology* 17:1. 10.1186/s12951-018-0433-433 30612562 PMC6322342

[B46] Lister R. Pelizzola M. Kida Y. S. Hawkins R. D. Nery J. R. Hon G. (2011). Hotspots of aberrant epigenomic reprogramming in human induced pluripotent stem cells. *Nature* 471 68–73. 10.1038/nature09798 21289626 PMC3100360

[B47] Liu X. Li Q. Niu X. Hu B. Chen S. Song W. (2017). Exosomes secreted from human-induced pluripotent stem cell-derived mesenchymal stem cells prevent osteonecrosis of the femoral head by promoting angiogenesis. *Int. J. Biol. Sci.* 13 232–244. 10.7150/ijbs.16951 28255275 PMC5332877

[B48] Livshits M. A. Khomyakova E. Evtushenko E. G. Lazarev V. N. Kulemin N. A. Semina S. E. (2015). Isolation of exosomes by differential centrifugation: theoretical analysis of a commonly used protocol. *Sci. Rep.* 5:17319. 10.1038/srep17319 26616523 PMC4663484

[B49] Luarte A. Bátiz L. F. Wyneken U. Lafourcade C. (2016). Potential therapies by stem cell-derived exosomes in CNS diseases: focusing on the neurogenic niche. *Stem Cells Int.* 2016 1–16. 10.1155/2016/5736059 27195011 PMC4853949

[B50] Mi H. Muruganujan A. Thomas P. D. (2013). PANTHER in 2013: modeling the evolution of gene function, and other gene attributes, in the context of phylogenetic trees. *Nucleic Acids Res.* 41 D377–D386. 10.1093/nar/gks1118 23193289 PMC3531194

[B51] Mi H. Muruganujan A. Huang X. Ebert D. Mills C. Guo X. (2019). Protocol update for large-scale genome and gene function analysis with the PANTHER classification system (v.14.0). *Nat. Protoc.* 14 703–721. 10.1038/s41596-019-0128-8 30804569 PMC6519457

[B52] Miller A. M. Shah R. H. Pentsova E. I. Pourmaleki M. Briggs S. Distefano N. (2019). Tracking tumour evolution in glioma through liquid biopsies of cerebrospinal fluid. *Nature* 565 654–658. 10.1038/s41586-019-0882-883 30675060 PMC6457907

[B53] Mol E. A. Goumans M.-J. Doevendans P. A. Sluijter J. P. G. Vader P. (2017). Higher functionality of extracellular vesicles isolated using size-exclusion chromatography compared to ultracentrifugation. *Nanomedicine* 13 2061–2065. 10.1016/j.nano.2017.03.011 28365418

[B54] Moreno-Gonzalo O. Fernandez-Delgado I. Sanchez-Madrid F. (2018). Post-translational add-ons mark the path in exosomal protein sorting. *Cell. Mol. Life Sci.* 75 1–19. 10.1007/s00018-017-2690-y 29080091 PMC11105655

[B55] Munoz J. Low T. Y. Kok Y. J. Chin A. Frese C. K. Ding V. (2014). The quantitative proteomes of human-induced pluripotent stem cells and embryonic stem cells. *Mol. Syst. Biol.* 7 550–550. 10.1038/msb.2011.84 22108792 PMC3261715

[B56] Narsinh K. H. Sun N. Sanchez-Freire V. Lee A. S. Almeida P. Hu S. (2011). Single cell transcriptional profiling reveals heterogeneity of human induced pluripotent stem cells. *J. Clin. Invest.* 121 1217–1221. 10.1172/JCI44635 21317531 PMC3049389

[B57] Noerholm M. Balaj L. Limperg T. Salehi A. Zhu L. D. Hochberg F. H. (2012). RNA expression patterns in serum microvesicles from patients with glioblastoma multiforme and controls. *BMC Cancer* 12:22. 10.1186/1471-2407-12-22 22251860 PMC3329625

[B58] Nordin J. Z. Lee Y. Vader P. Mäger I. Johansson H. J. Heusermann W. (2015). Ultrafiltration with size-exclusion liquid chromatography for high yield isolation of extracellular vesicles preserving intact biophysical and functional properties. *Nanomedicine* 11 879–883. 10.1016/j.nano.2015.01.003 25659648

[B59] Oeyen E. Van Mol K. Baggerman G. Willems H. Boonen K. Rolfo C. (2018). Ultrafiltration and size exclusion chromatography combined with asymmetrical-flow field-flow fractionation for the isolation and characterisation of extracellular vesicles from urine. *J. Extracell. Vesicles* 7:1490143. 10.1080/20013078.2018.1490143 29988836 PMC6032024

[B60] Oh M. Lee J. Kim Y. Rhee W. Park J. (2018). Exosomes derived from human induced pluripotent stem cells ameliorate the aging of skin fibroblasts. *Int. J. Mol. Sci.* 19:1715. 10.3390/ijms19061715 29890746 PMC6032439

[B61] Ohi Y. Qin H. Hong C. Blouin L. Polo J. M. Guo T. (2011). Incomplete DNA methylation underlies a transcriptional memory of somatic cells in human iPS cells. *Nat. Cell Biol.* 13 541–549. 10.1038/ncb2239 21499256 PMC3987913

[B62] Onódi Z. Pelyhe C. Terézia Nagy C. Brenner G. B. Almási L. Kittel Á. (2018). Isolation of high-purity extracellular vesicles by the combination of iodixanol density gradient ultracentrifugation and bind-elute chromatography from blood plasma. *Front. Physiol.* 9:1479. 10.3389/fphys.2018.01479 30405435 PMC6206048

[B63] Osafune K. Caron L. Borowiak M. Martinez R. J. Fitz-Gerald C. S. Sato Y. (2008). Marked differences in differentiation propensity among human embryonic stem cell lines. *Nat. Biotechnol.* 26 313–315. 10.1038/nbt1383 18278034

[B64] Otake K. Kamiguchi H. Hirozane Y. (2019). Identification of biomarkers for amyotrophic lateral sclerosis by comprehensive analysis of exosomal mRNAs in human cerebrospinal fluid. *BMC Med. Genomics* 12:7. 10.1186/s12920-019-0473-z 30630471 PMC6329125

[B65] Pan B. T. Teng K. Wu C. Adam M. Johnstone R. M. (1985). Electron microscopic evidence for externalization of the transferrin receptor in vesicular form in sheep reticulocytes. *J. Cell Biol.* 101 942–948. 10.1083/jcb.101.3.942 2993317 PMC2113705

[B66] Peinado H. Alec̀kovič M. Lavotshkin S. Matei I. Costa-Silva B. Moreno-Bueno G. (2012). Melanoma exosomes educate bone marrow progenitor cells toward a pro-metastatic phenotype through MET. *Nat. Med.* 18 883–891. 10.1038/nm.2753 22635005 PMC3645291

[B67] Phanstiel D. H. Brumbaugh J. Wenger C. D. Tian S. Probasco M. D. Bailey D. J. (2011). Proteomic and phosphoproteomic comparison of human ES and iPS cells. *Nat. Methods* 8 821–827. 10.1038/nmeth.1699 21983960 PMC3432645

[B68] Pozzi D. Ban J. Iseppon F. Torre V. (2017). An improved method for growing neurons: comparison with standard protocols. *J. Neurosci. Methods* 280 1–10. 10.1016/j.jneumeth.2017.01.013 28137433

[B69] Ragusa M. Barbagallo C. Cirnigliaro M. Battaglia R. Brex D. Caponnetto A. (2017). Asymmetric RNA distribution among cells and their secreted exosomes: biomedical meaning and considerations on diagnostic applications. *Front. Mol. Biosci.* 4:66. 10.3389/fmolb.2017.00066 29046875 PMC5632685

[B70] Raposo G. Nijman H. W. Stoorvogel W. Liejendekker R. Harding C. V. Melief C. J. (1996). B lymphocytes secrete antigen-presenting vesicles. *J. Exp. Med.* 183 1161–1172. 10.1084/jem.183.3.1161 8642258 PMC2192324

[B71] Ratajczak J. Miekus K. Kucia M. Zhang J. Reca R. Dvorak P. (2006). Embryonic stem cell-derived microvesicles reprogram hematopoietic progenitors: evidence for horizontal transfer of mRNA and protein delivery. *Leukemia* 20 847–856. 10.1038/sj.leu.2404132 16453000

[B72] Raz A. Barzilai R. Spira G. Inbar M. (1978). Oncogenicity and immunogenicity associated with membranes isolated from cell-free ascites fluid of lymphoma-bearing mice. *Cancer Res.* 38 2480–2485. 667843

[B73] Rekker K. Saare M. Roost A. M. Kubo A.-L. Zarovni N. Chiesi A. (2014). Comparison of serum exosome isolation methods for microRNA profiling. *Clin. Biochem.* 47 135–138. 10.1016/j.clinbiochem.2013.10.020 24183884

[B74] Rood I. M. Deegens J. K. J. Merchant M. L. Tamboer W. P. M. Wilkey D. W. Wetzels J. F. M. (2010). Comparison of three methods for isolation of urinary microvesicles to identify biomarkers of nephrotic syndrome. *Kidney Int.* 78 810–816. 10.1038/ki.2010.262 20686450

[B75] Sakisaka T. (2005). Cell adhesion molecules in the CNS. *J. Cell Sci.* 118 5407–5410. 10.1242/jcs.02672 16306219

[B76] Segura E. Amigorena S. Théry C. (2005a). Mature dendritic cells secrete exosomes with strong ability to induce antigen-specific effector immune responses. *Blood Cells. Mol. Dis.* 35 89–93. 10.1016/j.bcmd.2005.05.003 15990342

[B77] Segura E. Nicco C. Lombard B. Véron P. Raposo G. Batteux F. (2005b). ICAM-1 on exosomes from mature dendritic cells is critical for efficient naive T-cell priming. *Blood* 106 216–223. 10.1182/blood-2005-01-0220 15790784

[B78] Shelke G. V. Lässer C. Gho Y. S. Lötvall J. (2014). Importance of exosome depletion protocols to eliminate functional and RNA-containing extracellular vesicles from fetal bovine serum. *J. Extracell. Vesicles* 3:24783. 10.3402/jev.v3.24783 25317276 PMC4185091

[B79] Shimaoka M. Kawamoto E. Gaowa A. Okamoto T. Park E. (2019). Connexins and integrins in exosomes. *Cancers* 11:106. 10.3390/cancers11010106 30658425 PMC6356207

[B80] Sork H. Corso G. Krjutskov K. Johansson H. J. Nordin J. Z. Wiklander O. P. B. (2018). Heterogeneity and interplay of the extracellular vesicle small RNA transcriptome and proteome. *Sci. Rep.* 8:10813. 10.1038/s41598-018-28485-28489 30018314 PMC6050237

[B81] Stranska R. Gysbrechts L. Wouters J. Vermeersch P. Bloch K. Dierickx D. (2018). Comparison of membrane affinity-based method with size-exclusion chromatography for isolation of exosome-like vesicles from human plasma. *J. Transl. Med.* 16:1. 10.1186/s12967-017-1374-6 29316942 PMC5761138

[B82] Sunkara V. Kim C.-J. Park J. Woo H.-K. Kim D. Ha H. K. (2019). Fully automated, label-free isolation of extracellular vesicles from whole blood for cancer diagnosis and monitoring. *Theranostics* 9 1851–1863. 10.7150/thno.32438 31037143 PMC6485293

[B83] Szostak N. Royo F. Rybarczyk A. Szachniuk M. Blazewicz J. del Sol A. (2014). Sorting signal targeting mRNA into hepatic extracellular vesicles. *RNA Biol.* 11 836–844. 10.4161/rna.29305 24921245 PMC4179958

[B84] Takov K. Yellon D. M. Davidson S. M. (2019). Comparison of small extracellular vesicles isolated from plasma by ultracentrifugation or size-exclusion chromatography: yield, purity and functional potential. *J. Extracell. Vesicles* 8:1560809. 10.1080/20013078.2018.1560809 30651940 PMC6327926

[B85] Tauro B. J. Greening D. W. Mathias R. A. Ji H. Mathivanan S. Scott A. M. (2012). Comparison of ultracentrifugation, density gradient separation, and immunoaffinity capture methods for isolating human colon cancer cell line LIM1863-derived exosomes. *Methods* 56 293–304. 10.1016/j.ymeth.2012.01.002 22285593

[B86] Taylor D. D. Zacharias W. Gercel-Taylor C. (2011). “Exosome Isolation for Proteomic Analyses and RNA Profiling,” in *Serum/Plasma Proteomics*, eds Simpson R. J. Greening D. W. (Totowa, NJ: Humana Press), 235–246. 10.1007/978-1-61779-068-3_15 21468952

[B87] Théry C. Amigorena S. Raposo G. Clayton A. (2006). Isolation and characterization of exosomes from cell culture supernatants and biological fluids. *Curr. Protoc. Cell Biol.* Chapter 3:Unit 3.22. 10.1002/0471143030.cb0322s30 18228490

[B88] Théry C. Boussac M. Véron P. Ricciardi-Castagnoli P. Raposo G. Garin J. (2001). Proteomic analysis of dendritic cell-derived exosomes: a secreted subcellular compartment distinct from apoptotic vesicles. *J. Immunol.* 166 7309–7318. 10.4049/jimmunol.166.12.7309 11390481

[B89] Thompson A. G. Gray E. Heman-Ackah S. M. Mäger I. Talbot K. Andaloussi S. E. (2016). Extracellular vesicles in neurodegenerative disease - pathogenesis to biomarkers. *Nat. Rev. Neurol.* 12 346–357. 10.1038/nrneurol.2016.68 27174238

[B90] Thompson A. G. Gray E. Mager I. Fischer R. Thézénas M.-L. Charles P. D. (2018). UFLC-Derived CSF extracellular vesicle origin and proteome. *Proteomics* 18:e1800257. 10.1002/pmic.201800257 30411858

[B91] Trams E. G. Lauter C. J. Salem N. Heine U. (1981). Exfoliation of membrane ecto-enzymes in the form of micro-vesicles. *Biochim. Biophys. Acta* 645 63–70. 10.1016/0005-2736(81)90512-5 6266476

[B92] Usman W. M. Pham T. C. Kwok Y. Y. Vu L. T. Ma V. Peng B. (2018). Efficient RNA drug delivery using red blood cell extracellular vesicles. *Nat. Commun.* 9:2359. 10.1038/s41467-018-04791-4798 29907766 PMC6004015

[B93] Utsugi-Kobukai S. Fujimaki H. Hotta C. Nakazawa M. Minami M. (2003). MHC class I-mediated exogenous antigen presentation by exosomes secreted from immature and mature bone marrow derived dendritic cells. *Immunol. Lett.* 89 125–131. 10.1016/s0165-2478(03)00128-7 14556969

[B94] Villarroya-Beltri C. Baixauli F. Gutiérrez-Vázquez C. Sánchez-Madrid F. Mittelbrunn M. (2014). Sorting it out: regulation of exosome loading. *Semin. Cancer Biol.* 28 3–13. 10.1016/j.semcancer.2014.04.009 24769058 PMC4640178

[B95] Wang G. Dinkins M. He Q. Zhu G. Poirier C. Campbell A. (2012). Astrocytes secrete exosomes enriched with proapoptotic ceramide and prostate apoptosis response 4 (PAR-4): potential mechanism of apoptosis induction in Alzheimer disease (AD). *J. Biol. Chem.* 287 21384–21395. 10.1074/jbc.M112.340513 22532571 PMC3375560

[B96] Wang X. Jauch R. (2014). OCT4: a penetrant pluripotency inducer. *Cell Regen.* 3:6. 10.1186/2045-9769-3-6 25408885 PMC4230516

[B97] Ward C. M. Barrow K. M. Stern P. L. (2004). Significant variations in differentiation properties between independent mouse ES cell lines cultured under defined conditions. *Exp. Cell Res.* 293 229–238. 10.1016/j.yexcr.2003.10.017 14729460

[B98] Wei Z. Batagov A. O. Carter D. R. F. Krichevsky A. M. (2016). Fetal bovine serum RNA interferes with the cell culture derived extracellular RNA. *Sci. Rep.* 6:31175. 10.1038/srep31175 27503761 PMC4977539

[B99] Welton J. L. Loveless S. Stone T. von Ruhland C. Robertson N. P. Clayton A. (2017). Cerebrospinal fluid extracellular vesicle enrichment for protein biomarker discovery in neurological disease; multiple sclerosis. *J. Extracell. Vesicles* 6:1369805. 10.1080/20013078.2017.1369805 28959386 PMC5614217

[B100] Welton J. L. Webber J. P. Botos L.-A. Jones M. Clayton A. (2015). Ready-made chromatography columns for extracellular vesicle isolation from plasma. *J. Extracell. Vesicles* 4:27269. 10.3402/jev.v4.27269 25819214 PMC4376847

[B101] Wolf P. (1967). The nature and significance of platelet products in human plasma. *Br. J. Haematol.* 13 269–288. 10.1111/j.1365-2141.1967.tb08741.x 6025241

[B102] Yamada T. Inoshima Y. Matsuda T. Ishiguro N. (2012). Comparison of methods for isolating exosomes from bovine milk. *J. Vet. Med. Sci.* 74 1523–1525. 10.1292/jvms.12-0032 22785357

[B103] Yuan A. Farber E. L. Rapoport A. L. Tejada D. Deniskin R. Akhmedov N. B. (2009). Transfer of microRNAs by embryonic stem cell microvesicles. *PLoS One* 4:e4722. 10.1371/journal.pone.0004722 19266099 PMC2648987

[B104] Yuana Y. Levels J. Grootemaat A. Sturk A. Nieuwland R. (2014). Co-isolation of extracellular vesicles and high-density lipoproteins using density gradient ultracentrifugation. *J. Extracell. Vesicles* 3:23262. 10.3402/jev.v3.23262 25018865 PMC4090368

[B105] Zhang Z. Wang C. Li T. Liu Z. Li L. (2014). Comparison of ultracentrifugation and density gradient separation methods for isolating Tca8113 human tongue cancer cell line-derived exosomes. *Oncol. Lett.* 8 1701–1706. 10.3892/ol.2014.2373 25202395 PMC4156197

[B106] Zhou J. Ghoroghi S. Benito-Martin A. Wu H. Unachukwu U. J. Einbond L. S. (2016). Characterization of induced pluripotent stem cell microvesicle genesis, morphology and pluripotent content. *Sci. Rep.* 6:19743. 10.1038/srep19743 26797168 PMC4726265

[B107] Zhu Y. Wang Y. Zhao B. Niu X. Hu B. Li Q. (2017). Comparison of exosomes secreted by induced pluripotent stem cell-derived mesenchymal stem cells and synovial membrane-derived mesenchymal stem cells for the treatment of osteoarthritis. *Stem Cell Res. Ther.* 8:64. 10.1186/s13287-017-0510-9 28279188 PMC5345222

[B108] Zitvogel L. Regnault A. Lozier A. Wolfers J. Flament C. Tenza D. (1998). Eradication of established murine tumors using a novel cell-free vaccine: dendritic cell-derived exosomes. *Nat. Med.* 4 594–600. 10.1038/nm0598-594 9585234

