# Supplementary figures and images for: Considerations and Implications in the Purification of Extracellular Vesicles – A Cautionary Tale
Source: Front Neurosci. 2019 Oct 18;13:1067. doi: 10.3389/fnins.2019.01067 (PMC6813730; doi:10.3389/fnins.2019.01067)

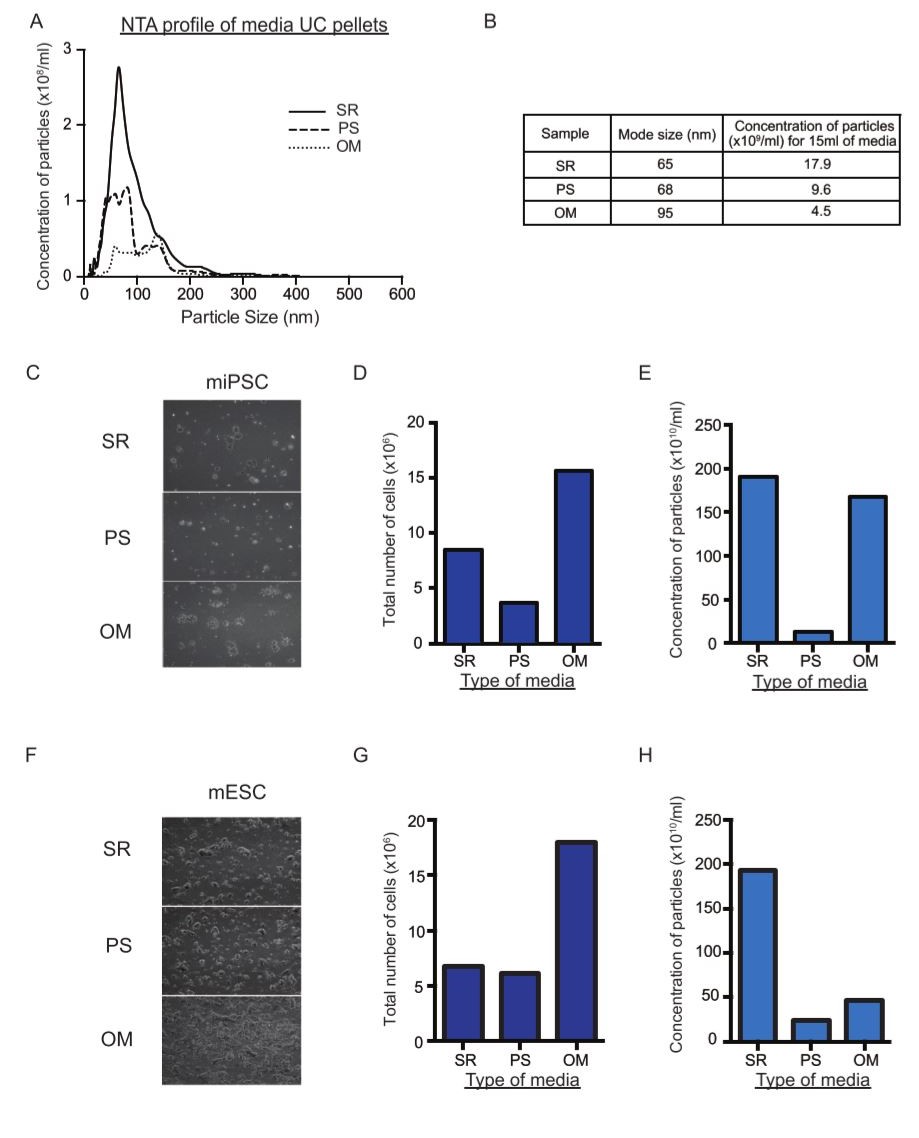

Supplement: FIGURE S1 — Alternative types of serum-depleted media for EV collection from stem cells. (A) NTA size distribution profiles of UC pellets after centrifugation of the three types of media: KOSR-supplemented stem cell media (SR), Pre-spun stem cell media (PS) and serum-free media/OptiMEM (OM). (B) Table showing the mode size (nm) and concentration of particles (x109/ml) detected in the UC pellet of each media type. Representative bright-field images of mouse iPSCs (C) and mouse ESCs (F) after 48 h culture in SR, PS, or OM. Graphs comparing the total number of viable cells in the miPSCs (D) and mESCs (E) cultures at CM collection. Graphs comparing the total concentration of particles (x1010/ml) detected by NTA in the UC pellet of miPSCs (G) and mESCs (H) in the three different media types. [file Image_1.JPEG]

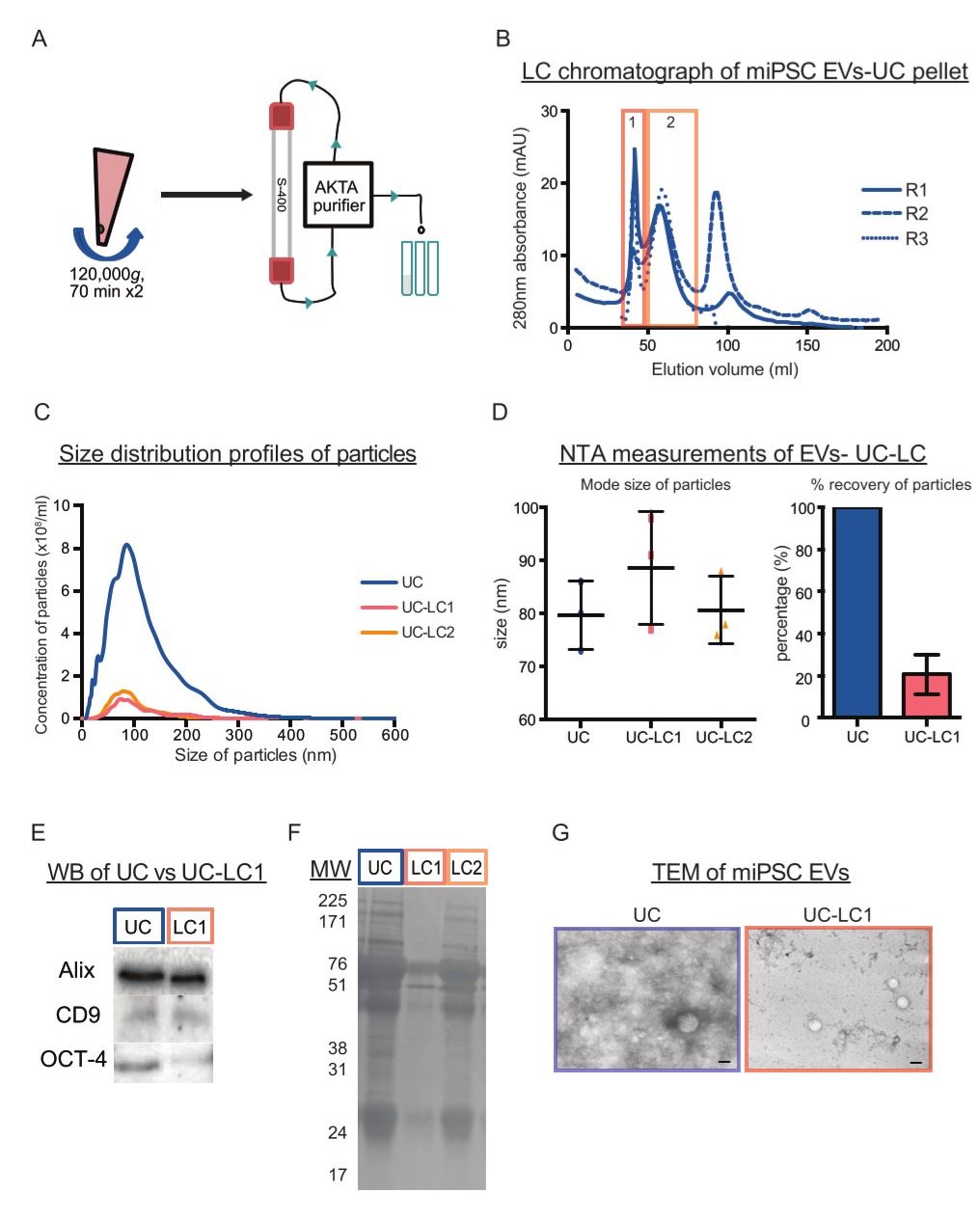

Supplement: FIGURE S2 — LC fractionation of UC pellets reveals co-precipitation of non-vesicular proteins with EVs in the UC pellet. (A) Schematic outline of the LC fractionation protocol of UC pellets. (B) LC chromatograph showing the 280 nm absorbance of the elution course from the LC column for three replicate samples (R1–R3). The first fraction (pink box) corresponds to the region where EVs elute. The second fraction (orange box) shows the appearance of another peak after the EVs. (C) NTA size distribution profiles of particles in the original UC sample (blue), and in the first (box 1; UC-LC1) and second (box 2; UC-LC2) LC fractions. (D) Graph on the left shows the mode size (nm) of particles in the original UC pellet, UC-LC1 and UC-LC2 (n = 3, bar represents mean ± SD). Graph on the right shows the percentage of particles detected in the UC pellet versus UC-LC1 (n = 3, bar represents mean ± SD). (E) Representative western blots for EV (Alix and CD9) and pluripotency (OCT4) markers when loading the same amount of particles from the UC pellet and UC-LC1 fraction. (F) Total protein staining of the UC pellet, UC-LC1 and UC-LC2. (G) Representative TEM images of the UC pellet and UC-LC1. Here the UC-LC1 sample appears to have a decreased background as compared to UC. The scale bar corresponds to 100 nm. [file Image_2.JPEG]

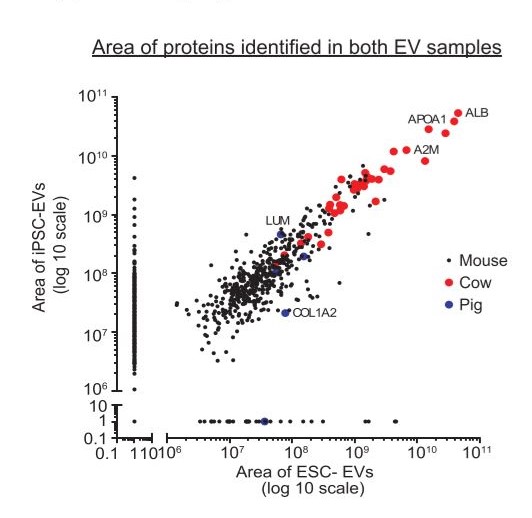

Supplement: FIGURE S3 — Detection of non-mouse proteins in mouse derived EVs. Scatter plot showing the areas of proteins identified in the preliminary analysis of iPSC- and ESC-EV samples. Proteins from mouse (black dots), cow (red dots) and pig (blue dots) are indicated. [file Image_3.JPEG]

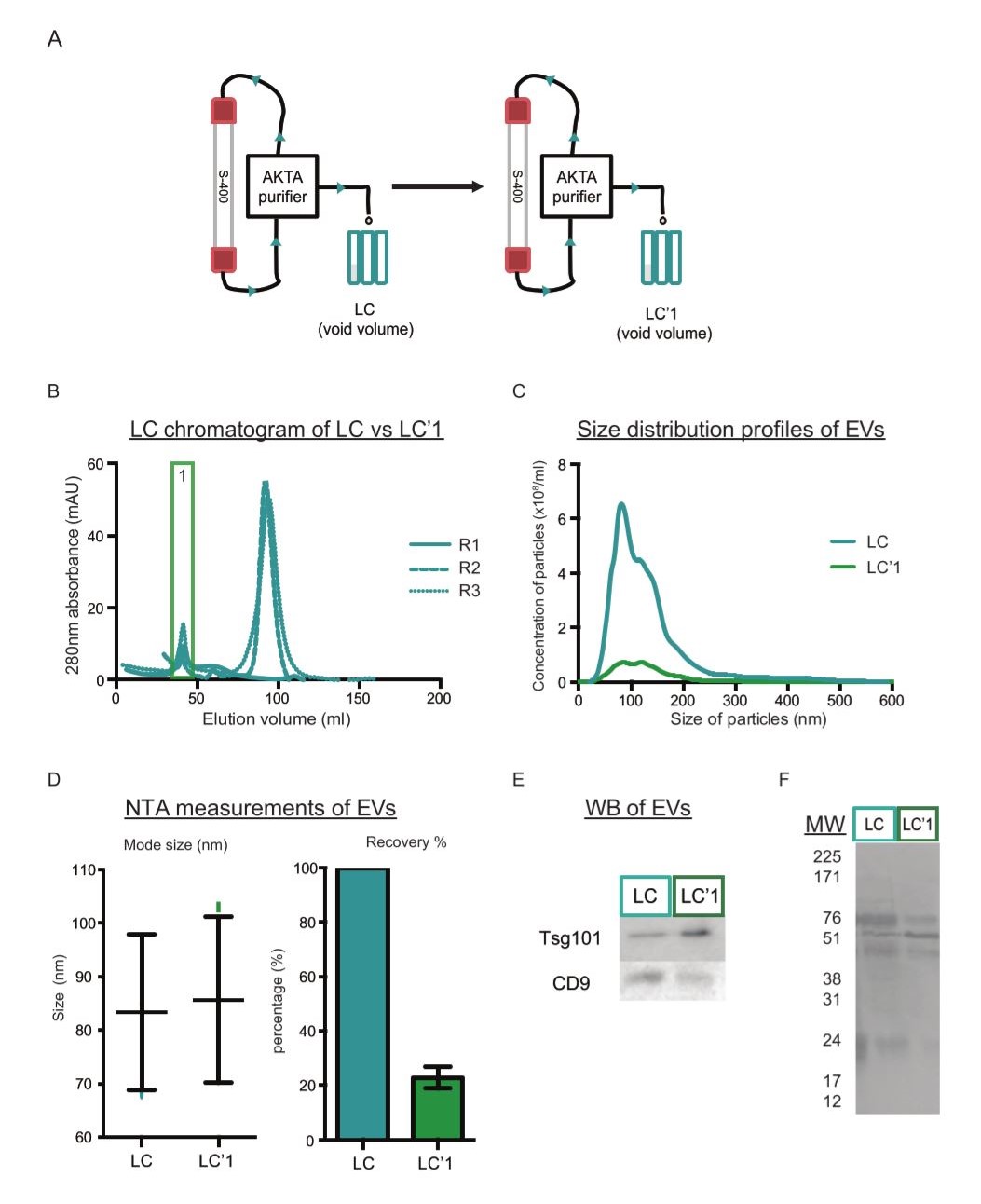

Supplement: FIGURE S4 — Sequential LC fraction improves EV purity marginally. (A) Schematic outline of the sequential LC fractionation set-up. (B) LC chromatograph showing the 280 nm absorbance of the elution course from the LC column for three replicate samples (R1–R3). The first fraction (green box) corresponds to the region where EVs elute. (C) NTA size distribution profiles of particles in the first LC sample versus the second LC run (LC′1). (D) On the left, the mode size of particles in the original LC sample and the LC′1 sample appears to be similar (n = 3). On the right, graph showing overall percentage of particles recovered as compared to the input material. (E) Representative western blotting pictures when loading the same amount of particles for LC and LC’1. (F) Total protein staining of the membrane with LC and LC’1 samples. [file Image_4.JPEG]
